# Autophagy regulates UBC9 levels during viral-mediated tumorigenesis

**DOI:** 10.1371/journal.ppat.1006262

**Published:** 2017-03-02

**Authors:** Domenico Mattoscio, Chiara Casadio, Claudia Miccolo, Fausto Maffini, Andrea Raimondi, Carlo Tacchetti, Tarik Gheit, Marta Tagliabue, Viviana E. Galimberti, Francesca De Lorenzi, Michael Pawlita, Fausto Chiesa, Mohssen Ansarin, Massimo Tommasino, Susanna Chiocca

**Affiliations:** 1 European Institute of Oncology, Department of Experimental Oncology, Milan, Italy; 2 European Institute of Oncology, Department of Pathology, Milan, Italy; 3 Experimental Imaging Center, IRCCS San Raffaele Scientific Institute, Milan, Italy; 4 Department of Experimental Medicine, University of Genova, Genova, Italy; 5 Infections and Cancer Biology Group, International Agency for Research on Cancer, Lyon, France; 6 European Institute of Oncology, Division of Otolaryngology and Head and Neck Surgery, Milan, Italy; 7 European Institute of Oncology Senology Division, Milan, Italy; 8 European Institute of Oncology, Plastic Surgery Department, Milan, Italy; 9 Division of Molecular Diagnostics of Oncogenic Infections, Research Program Infection, Inflammation and Cancer, German Cancer Research Center, Heidelberg, Germany; University of North Carolina at Chapel Hill, UNITED STATES

## Abstract

UBC9, the sole E2-conjugating enzyme required for SUMOylation, is a key regulator of essential cellular functions and, as such, is frequently altered in cancers. Along these lines, we recently reported that its expression gradually increases during early stages of human papillomavirus (HPV)-mediated cervical lesions transformation. However, a better understanding of how UBC9 is exploited by transforming viral oncoproteins is still needed. In the present study, we show that in human samples HPV drives UBC9 up-regulation also in very early steps of head and neck tumorigenesis, pointing to the important role for UBC9 in the HPV-mediated carcinogenic program. Moreover, using HPV-infected pre-cancerous tissues and primary human keratinocytes as the natural host of the virus, we investigate the pathological meaning and the cellular mechanisms responsible for UBC9 de-regulation in an oncoviral context. Our results show that UBC9 overexpression is promoted by transforming viral proteins to increase host cells’ resistance to apoptosis. In addition, ultrastuctural, pharmacological and genetic approaches crucially unveil that UBC9 is physiologically targeted by autophagy in human cells. However, the presence of HPV E6/E7 oncoproteins negatively impacts the autophagic process through selective inhibition of autophagosome-lysosome fusion, finally leading to p53 dependent UBC9 accumulation during viral-induced cellular transformation. Therefore, our study elucidates how UBC9 is manipulated by HPV oncoproteins, details the physiological mechanism by which UBC9 is degraded in cells, and identifies how HPV E6/E7 impact on autophagy. These findings point to UBC9 and autophagy as novel hallmarks of HPV oncogenesis, and open innovative avenues towards the treatment of HPV-related malignancies.

## Author summary

High risk HPV is the primary cause of cervical cancer and in recent years a clear role for the virus in other anogenital malignancies, in head and neck and in nonmelanoma skin cancers is emerging. Cellular transformation is mediated by the viral oncoproteins E6 and E7 that have the ability to target several pathways. We recently demonstrated that UBC9, the key enzyme of the Small Ubiquitin-like Modifier (SUMO) pathway, is up-regulated during pre-cancerous and cancerous stages of cervical carcinogenesis. In this study, we elucidate the pathological impact and the cellular mechanisms responsible for UBC9 accumulation promoted by HPV in cervical and head and neck pre-cancerous tissues. Indeed, we show that UBC9 is physiologically degraded in cells by autophagy, and that the expression of HPV oncoproteins impairs autophagy finally leading to UBC9 accumulation and apoptosis resistance of infected cells. Our results therefore identify UBC9 and autophagy as important co-factors to prime early stages of HPV-mediated tumorigenesis, and pinpoint novel therapeutic strategies for treatment of carcinogenic HPV infections.

## Introduction

Oncovirus infection represents one of the most common agents involved in cancer pathogenesis [1]. In particular, human papilloma virus (HPV) cancer burden is still very high despite the advent of the vaccines, accounting for more than 600,000 new cancer cases worldwide [2]. Indeed, even though cervical cancer is the most common cancer caused by HPV [3], a significant role of this virus family in other anogenital malignancies, in nonmelanoma skin cancers, and in head and neck cancers (HNC) subtypes is clearly emerging [2,4]. Although the overall incidence of HNC is decreasing in developed countries due to raising awareness of tobacco and alcohol as risk factors for human carcinogenesis, the proportion of oropharyngeal carcinomas (OPSCC), the most frequently HPV-related [5], has been steadily increasing in the USA and Europe [6]. Therefore, a better understanding of HPV tumorigenesis is of paramount importance.

The E6 and E7 oncoproteins from high risk HPV types, mainly promoting the degradation of p53 and pRb inactivation and degradation [7,8] are the key culprits of malignant transformation. In addition, the viral oncoproteins are able to interact with a large number of cellular proteins altering their normal function and facilitating cellular transformation.

SUMOylation (Small Ubiquitin-like MOdifier) is a key post-translational modification (PTM) that critically regulates a plethora of cellular functions [9,10]. The SUMO pathway modulates the activity of target proteins through reversible conjugation of one of the different SUMO paralogs (SUMO1 and the nearly identical SUMO2 and 3 in humans [11]) by an ubiquitin-like pathway that involves the sequential action of different enzymes: SUMO-activating (SAE1/SAE2), SUMO-conjugating (UBC9), several SUMO ligases, and SUMO proteases [12]. UBC9, in particular, is the key protein of the SUMO machinery since it is the unique E2 conjugating enzyme and it can directly interact and transfer SUMO moiety on target proteins [13]. Therefore, altered UBC9 levels alone may modify cellular SUMOylation pattern strikingly affecting a wide range of cellular activities. Moreover, in addition to its crucial involvement in SUMOylation, UBC9 may also function as a molecular chaperone [14], protein stabilizer [15], or as transcriptional [16] and miRNAs regulator [17]. Accordingly to its pleiotropic role, UBC9 is frequently targeted by numerous viral proteins [18–20] and is often altered in cancers, substantially contributing to the development of human malignancies (see [21] for a recent review). Indeed, recent evidence pinpoints to UBC9 as an important enzyme in human tumorigenesis since its selective targeting may easily subvert a variety of physiological pathway to support cancer growth. Namely, UBC9 overexpression dampens apoptosis in ovarian carcinoma [22], melanoma [23], and glioma [24], and enhances tumor growth and aggressiveness in breast [17] and colorectal cancers [25]. Along these lines, in our primary study we reported that UBC9 overexpression also occurred in pre-cancerous stages of cervical cancer [26], suggesting an important role for UBC9 in viral-induced cellular transformation. However, the biological significance and the mechanisms involved in this event in HPV-induced carcinogenesis remain to be investigated.

Here, using a variety of *ex vivo* and *in vitro* models to dissect HPV-tumorigenesis, we show that UBC9 accumulation occurs through viral oncoproteins’ expression also in other HPV-driven cancers, i.e. premalignant stages of HNC, to increase infected cells resistance to apoptosis. Moreover, we identify autophagy as a physiological route of UBC9 degradation, and provide innovative insights into the mechanism of UBC9 accumulation promoted by E6/E7 oncoproteins, highlighting UBC9 and autophagy as novel players in HPV-tumorigenesis.

## Results

### UBC9 is up-regulated during HPV-mediated pre-malignant transformation

**Fig 1:**
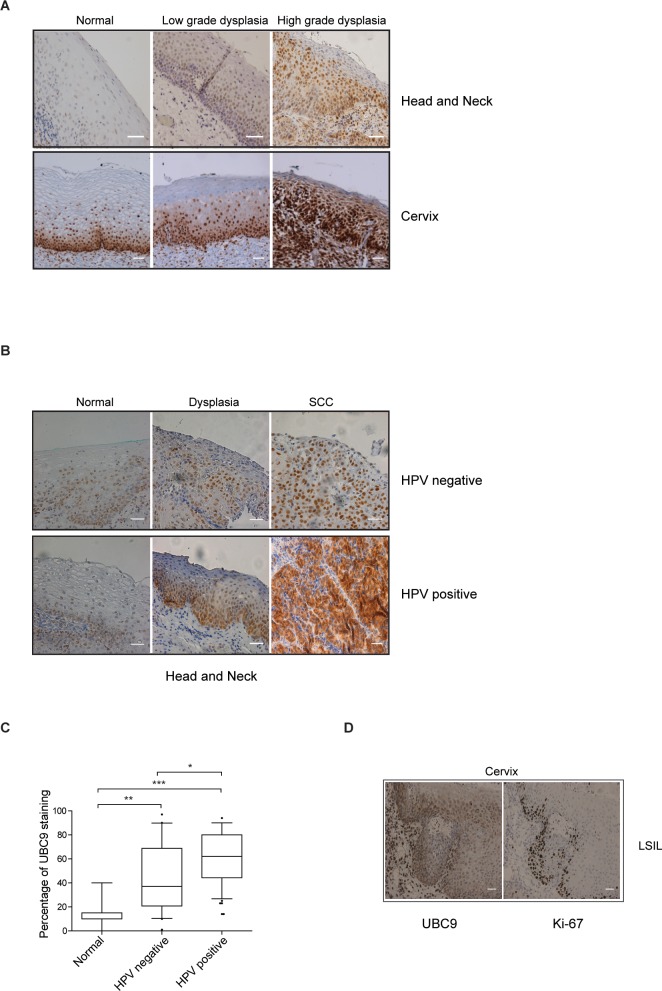
UBC9 is up-regulated during both cervical and head and neck tumorigenesis. **(A)** Representative IHC images of FFPE human normal, low grade or high grade dyplasia cervical or HN oropharyngeal tissues, as indicated, stained with anti-UBC9 antibody. Scale bars = 200nm. **(B)** Representative IHC images FFPE human normal, dysplastic or tumoral (squamous cell carcinoma-SCC) HN oropharyngeal HPV positive or negative tissues, as indicated, stained with anti-UBC9 antibody. Scale bars = 200nm. Samples were classified as HPV positive by DNA and RNA testing, as described in [88]. **(C)** Box and whisker plots showing the median and 10–90 percentiles of percentage of UBC9 positivity in HPV negative and HPV positive dysplastic and tumoral (SCC) HN oropharyngeal specimens. n = 20 (HPV negative) and 57 (HPV positive) different tissues. Outliers are shown by black circles. *P<0.05, **P < 0.001; ***P < 0.0001(one-way ANOVA followed by Tukey post hoc test). **(D)** Representative IHC images of FFPE human LSIL cervical tissue stained with anti-UBC9 or anti-Ki-67 antibodies, as indicated. Scale bars = 200nm.

To analyze whether UBC9 up-regulation was relevant for HPV-mediated carcinogenesis, we monitored its levels by immunohistochemistry (IHC) in a cohort of both HN and cervical formalin-fixed and paraffin-embedded (FFPE) pre-malignant lesions. In HN we observed weak UBC9 staining in normal tissues that increased in low-grade dysplasia and even higher in high-grade dysplastic tissues (Fig 1A). Likewise, representative IHC sections of human cervix confirmed a progressive up-regulation of UBC9 expression during lesion progression, corroborating our recent results ([26] and Fig 1A).

Since HNC could be both HPV-positive or HPV-negative [27], we stratified FFPE dysplastic and cancer HN tissues according to their HPV presence, and evaluated UBC9 positivity by quantification of UBC9 stained cells (Fig 1B and 1C). Remarkably, we found that HPV-expressing HN tissues are characterized by higher UBC9 positivity as compared to the HPV-negative counterparts, suggesting an intimate association between UBC9 and early steps of HPV-induced carcinogenesis.

In addition, we carried out IHC experiments staining cervical tissues with antibodies against UBC9 or the proliferation marker Ki-67. Our results clearly indicate that in the same tissue, normal or lesion, UBC9 and Ki-67 staining are not superimposable, suggesting that UBC9 expression is not a simple reflection of cellular proliferation (Fig 1D).

Collectively, these results indicate that UBC9 expression progressively increases during both cervical and HN cancer evolution and that HPV is an important driver of UBC9 up-regulation during HN tumorigenesis.

### UBC9 selectively drives accumulation of SUMO1-conjugated proteins during HPV-mediated transformation

**Fig 2:**
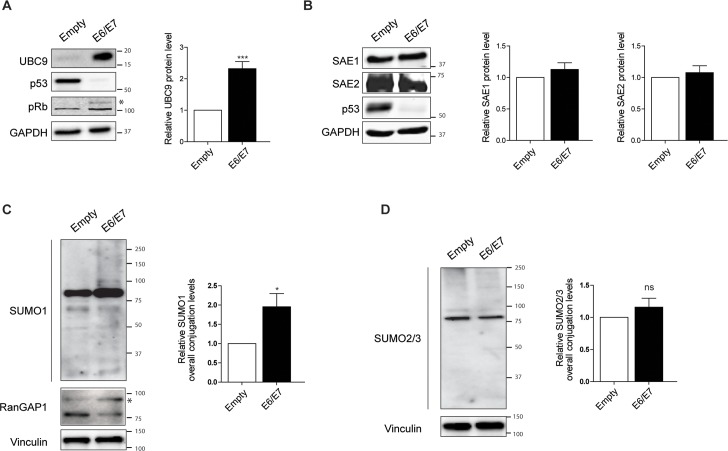
HPV16 E6/E7 selectively up-regulate UBC9 and SUMO1 conjugation. **(A)** and **(B)** Left: Representative WBs of HKs transduced with empty or HPV16 E6/E7 recombinant retroviral vectors. Successful E6/E7 infections were confirmed by p53 degradation and pRb inactivation. Asterisk marks the phosphorylated form of pRb. Right: Normalized protein bands intensities. Data are expressed as fold over the empty-transduced HKs. Bars represent means ± SEM of n = 8. ***P<0.0001 (paired-sample t-test). **(C)** and **(D)** Left: Representative WBs of HKs transduced with empty or HPV16 E6/E7 recombinant retroviral vectors, and blotted with antibodies against SUMO1, RanGAP1, and SUMO2/3, respectively. Asterisk marks SUMO1-modified RanGAP1. Right: Normalized SUMO1 and SUMO2/3 smear bands intensities, quantified as described in [91]. Data are expressed as fold over the empty-transduced HKs. Bars represent means ± SEM of n = 9. *P<0.05 (paired-sample t-test).

To assess how HPV de-regulates UBC9 expression we used primary human keratinocytes (HKs), the natural host of the virus, transduced with empty vectors or the two main viral oncogenes E6 and E7 from HPV16 [28]. Western blot (WB) analysis of transduced cells showed that, consistently with our *ex vivo* results (Fig 1 and [26]), HPV16 E6/E7 increased UBC9 protein expression as compared to empty control HKs (Fig 2A). Opposite, levels of the E1 SUMO Activating Enzyme subunits (SAE1 and SAE2) were completely unaffected (Fig 2B), suggesting a specific mechanism adopted by HPV16 E6/E7 to selectively up-regulate UBC9 levels.

Next, to fully understand the impact of HPV E6/E7 on the SUMO pathway, we investigated the overall SUMOylation status of transduced HKs. Our results showed that expression of HPV16 E6/E7 specifically promoted the accumulation of SUMO1, but not SUMO2/3, conjugated proteins in HKs (Fig 2C and 2D). In addition, WB analysis of RanGAP1, a well-known substrate of SUMO1 conjugation [29,30], showed a consistent increase of SUMO1- modified RanGAP1in E6/E7 HKs (Fig 2C), further pointing to the accumulation of SUMO1 conjugated species promoted by E6/E7.

**Fig 3:**
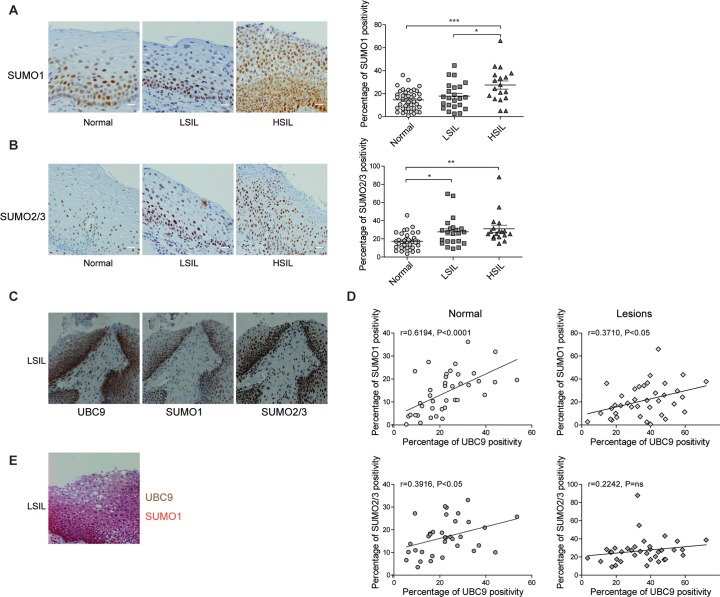
UBC9 preferentially promotes SUMO1 conjugation *ex vivo*. **(A)** and **(B)** Left: Representative IHC images of FFPE human normal, LSIL or HSIL cervical tissues stained with anti-SUMO1 or anti-SUMO2/3 antibodies, respectively. Right: Percentage of SUMO1-, or SUMO2/3-positive pixels, respectively, in FFPE human cervical tissues. Scale bars = 200nm. Data are expressed as percentage of positive plus strong positive stained pixels. The black lines represent the mean for each group. n = 37 (normal), 20 (LSIL), 20 (HSIL) different patients. *P<0.05; **P < 0.001; ***P < 0.0001(one-way ANOVA followed by Bonferroni post hoc test). **(C)** Representative IHC images of a FFPE human cervical tissue stained with anti-UBC9, anti-SUMO1 or anti-SUMO2/3 antibodies, respectively. **(D)** Top: Correlation between UBC9 and SUMO1 expression in paired normal (left) or lesion (right) tissues. n = 39 (normal), 38 (lesions) respectively. Bottom: Correlation between UBC9 and SUMO2/3 expression in paired normal (left) or lesion (right) tissues. n = 41 (normal), 38 (lesions) respectively. Spearman rank correlation was applied to analyze association between variables. Scattered plots with linear regression lines, Spearman r correlation coefficient, and p value are reported. **(E)** Representative IHC images of a FFPE human cervical tissue co-stained with anti-UBC9 (brown) and anti-SUMO1 (red). Note that almost all cells were both UBC9 and SUMO1 positive.

To confirm these *in vitro* results, we also evaluated both SUMO1 and SUMO2/3 positivity *ex vivo* in cervical FFPE specimens. Our computational quantification of IHC slides showed a differential pattern of SUMO paralogs expression in different stages of lesion progression. Specifically, even though both SUMO1 and SUMO2/3 positivity significantly increased in HSIL as compared to normal adjacent tissues, we observed that SUMO1 expression was increased during progression from LSIL (low-grade squamous intraepithelial lesion, or CIN1) to HSIL (high-grade squamous intraepithelial lesion or CIN2/3) (Fig 3A), while SUMO2/3 positivity was only affected during the transition between normal and LSIL cervical tissues (Fig 3B). To establish whether the preferential SUMO1 conjugation along with UBC9 increase in our *in vitro* experiments also occurred in cervical lesions, we stained the same tissues with anti-UBC9, -SUMO1, or -SUMO2/3 antibodies, respectively. Representative IHC slides reported in Fig 3C showed a clear overlap between UBC9 and SUMO1 expression patterns, while SUMO2/3 positivity was also found in different cells as compared to UBC9 (mainly in the superficial zone of stratified epithelia). Therefore, these results suggested that SUMO1 conjugation is intimately associated with UBC9 expression also in human lesions. To further corroborate our data, we performed a correlation analysis between UBC9 -SUMO1 and UBC9-SUMO2/3 positivity in matched pairs of normal and cervical lesion tissues. We found significant correlation of UBC9 positivity with both SUMO1 and SUMO2/3 in normal tissues, while in cervical lesions UBC9 correlated only with SUMO1 (Fig 3D) in agreement with our previous results (Figs 2C and 3C). Finally, co-stain analysis of UBC9 and SUMO1 expression reported in Fig 3E again highlights how the expression pattern of the two proteins is completely superimposable in human cervical specimens.

Therefore, collectively our results indicated that the higher UBC9 expression is coupled with an increased accumulation of proteins modified by SUMO1, suggesting an UBC9 preferential SUMO1-specificity promoted by E6/E7s. Importantly, our IHC analysis shows, for the first time, a global picture of SUMO paralogs and UBC9 expression in human pre-cancerous specimens during the natural evolution of HPV-driven diseases.

### UBC9 upregulation is promoted by viral proteins to increase host cells resistance to apoptosis

**Fig 4:**
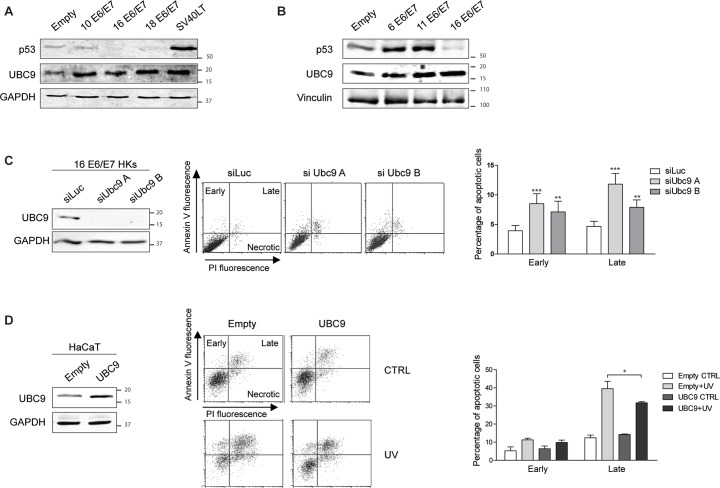
Transforming viral proteins similarly up-regulate UBC9 expression to extend keratinocytes life-span. **(A)** and **(B).** Left: Representative WB of HKs transduced with HPV E6/E7 from the indicated HPV types or SV40 large T antigen recombinant retroviruses. n = 4 different biological replicates. p53 accumulation is reported to check SV40 large T antigen expression. **(C)** Left: Representative WB analysis of HPV16 E6/E7 HKs after transfection with scramble (siLuc) or two different Ubc9 siRNAs. Middle: Representative cytometric dot plot of Annexin V/PI staining of siLuc or siUbc9s HPV16 E6/E7 HKs. y axes: Annexin V-FITC staining; x axes: PI staining. Quadrants of early, late and necrotic cells are reported. Right: Flow cytometric analysis of siLuc or siUbc9s cells apoptosis. Data are expressed as percentage of early and late apoptotic cells. Bars represent means ± SEM of n = 10 different biological replicates. **P < 0.001; ***P < 0.0001 (two-way ANOVA followed by Bonferroni post hoc test) compared to siLuc cells. **(D)** Left: Representative WB analysis of HaCaT cells after transfection with control (empty) or UBC9 protein. Middle: Representative cytometric dot plot of Annexin V/PI staining of empty or UBC9-HaCaT. y axes: Annexin V-FITC staining; x axes: PI staining. Quadrants of early, late and necrotic cells are reported. Right: Flow cytometric analysis of empty or UBC9-HaCaT apoptosis. Data are expressed as percentage of early and late apoptotic cells. Bars represent means ± SEM of n = 4 different replicates. *P < 0.05; (two-way ANOVA followed by Bonferroni post hoc test) compared to empty cells.

Then, we explored whether the ability to promote UBC9 overexpression was also shared by viral proteins other than HPV16. To this end, HKs were transduced with empty retroviral vector or E6/E7 proteins encoded by both low risk (LR) HPVs (mucosal HPV6 and HPV11, or cutaneous HPV10 types) or high risk (HR) HPVs (mucosal HPV16 and 18 types), and by the polyomavirus protein SV40 large T antigen, and UBC9 protein expression was analyzed by WB. The expression of the indicated constructs in HKs was verified by Real Time-quantitative PCR (RT-qPCR) (Fig A and Table A in S1 Text). We found that all the tested proteins upregulated UBC9 expression in HKs (Fig 4A and 4B), irrespectively of their tropism, oncogenic ability, or viral strain. Moreover, similarly to what obtained for UBC9, we observed that the expression of both LR and HR HPV E6/E7s selectively promoted the accumulation of SUMO1, but not SUMO2/3, conjugated proteins (Fig B in S1 Text), again suggesting an UBC9-driven SUMO1-specificity prompted by E6/E7s.

Since UBC9 overexpression dampens apoptosis in pathological contexts [22–24] and apoptosis resistance is a key feature of E6/E7-induced transformation [31], we then focused on apoptosis to test the biological meaning of UBC9 overexpression in viral-transformed cells. Flow cytometric analysis of HPV16 E6/E7 HKs transfected with control siRNA (luciferase -siLuc) or with two different siUBC9 oligos (Table B in S1 Text) showed that blockade of UBC9 expression considerably increased the percentage of both early (Annexin V+/PI-) and late (Annexin V+/PI+) apoptotic cells (Fig 4C), suggesting an important role for UBC9 in promoting apoptosis resistance of HPV16 E6/E7 HKs. To validate these results we overexpressed UBC9 in HPV-negative keratinocytes, HaCaT cells, and then we measured the percentage of apoptotic cells after stimulation with UV-irradiation. Consistently, we observed a significative reduction in UV-induced late apoptotic cells in UBC9-overexpressing HaCaT, thus confirming a protective role for UBC9 in delaying keratinocytes cell death (Fig 4D).

Therefore, these results suggest that diverse viral proteins up-regulate UBC9 expression to increase host cells resistance to apoptosis.

### p53 mediates UBC9 up-regulation triggered by HPV16 E6/E7

**Fig 5:**
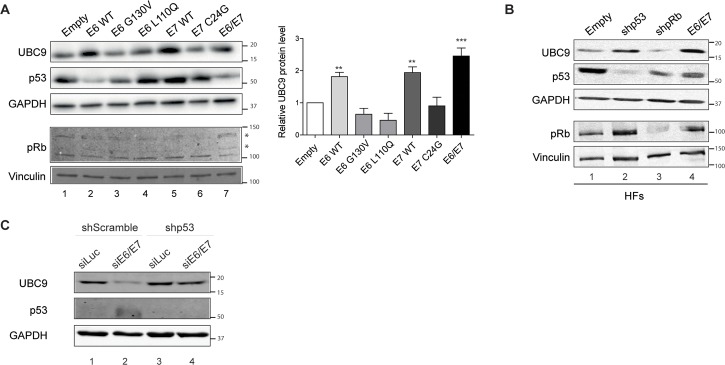
p53 mediate UBC9 accumulation triggered by HPV16 E6/E7. **(A)** Left: Representative WB of HKs transduced with the indicated recombinant retroviral vectors and analyzed by immunoblotting using anti- UBC9 antibody. Right: quantification of the protein bands intensities, standardized to GAPDH levels. Data are expressed as fold over the empty-transduced HKs. Bars represent means ± SEM of at least n = 9 biological replicates. *P<0.05; **P < 0.001; ***P < 0.0001(Kruskal–Wallis one-way ANOVA with Dunn’s post hoc test). p53 and pRb are reported as control for E6, E7, and mutants expression, respectively. Asterisks mark the phosphorylated forms of pRb. **(B)** Representative WB of HFs transduced with the indicated retroviral vectors and analyzed by immunoblotting using anti- UBC9, -p53, pRb antibodies. GAPDH and Vinculin are reported as loading control. n = 3 biological replicates. **(C)** Representative WB of Caski cells transduced with shScramble or shp53, as indicated, and treated with control siRNA (siLuc) or with siE6/E7 as described in Materials and Methods. Samples were analyzed by immunoblotting using anti- UBC9 and anti-p53antibodies. GAPDH is reported as loading control.

To gain more insights into the molecular mechanisms for UBC9 overexpression by HPV16 E6/E7, we evaluated the contribution of E6 and E7 individually in the regulation of UBC9 levels. WB analysis of HKs transduced with HPV16 E6 or E7 showed that both the oncoproteins similarly cooperate to stimulate UBC9 up-regulation (Fig 5A, lanes 1-2-5-7). Then, since the most important functions of high risk E6 and E7 are to promote p53 degradation and pRb inactivation [3] respectively, we analyzed the effect of two HPV16 E6 mutants (G130V and L110Q) unable to target p53 [32], and one HPV16 E7 mutant (C24G) incompetent to bind and inactivate pRb [33], in regulating UBC9 expression. In WB experiments we observed that G130V, L110Q and C24G mutations completely abolished the ability of E6 or E7 proteins to increase UBC9 protein levels (Fig 5A) in HKs, suggesting a p53/pRb-dependent mechanisms triggered by E6 and E7 to lead to UBC9 accumulation. To corroborate this hypothesis, we used primary human fibroblasts transduced with retroviral constructs to stably knock down p53 (shp53) or pRb (shpRb) proteins, or overexpress HPV16 E6/E7. In agreement with our previous results (Fig 5A) WB analysis revealed that, similarly to what observed with HPV16 E6/E7, UBC9 levels also increased in shp53 cells (Fig 5B, lane 2). Opposite, UBC9 levels were completely unaffected by shpRb in primary fibroblasts (Fig 5B, lane 3). Therefore, these results suggest that UBC9 overexpression by HPV16 E6/E7 occurs most likely through p53 inactivation, and that pRb inhibition appears additive since UBC9 induction is greater with E6/E7. In addition, to corroborate that E6-mediated UBC9 up-regulation occurred through p53 inactivation, we initially knocked down p53 and then we silenced E6/E7 in Caski cells, an HPV positive cell line derived from carcinoma of the uterine cervix that harbors normal levels of p53 mRNA [34]. Results reported in Fig 5C show that both E6/E7 siRNA and shp53 worked as expected (lanes 2 and 3), since p53 levels are restored upon E6/E7 siRNA (lane 2) in shScramble but not in shp53 cells (lane 4). Furthermore, WB analysis of UBC9 expression levels confirm the connection between increased UBC9 and p53 status. Accordingly with our previous results (Fig 5A and 5B), we found that UBC9 decreases when control Caski cells are treated with siE6/E7 (lane 2), increases in shp53 Caski (compare lane 3 to lane 1) and it is completely restored in siE6/E7 shp53 cells (compare lane 4 to lane 2), as in control cells (lane1). Therefore, this result indicates that E6/E7 silencing in Caski cells decreases UBC9 expression only when a functional p53 is present, confirming that E6/E7 alter UBC9 expression through p53.

### UBC9 is physiologically degraded by autophagy

**Fig 6:**
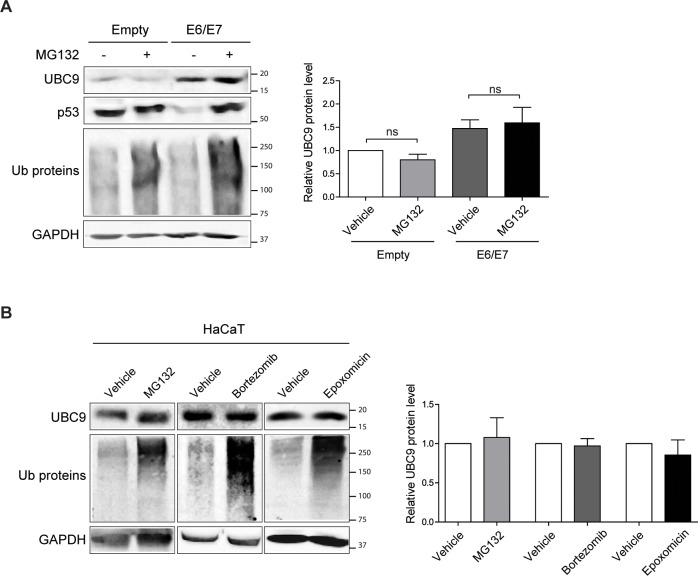
HPV16 E6/E7 disturb UBC9 degradation but not through the UPS. **(A)** Left: Representative WB of empty- or HPV16 E6/E7-transduced HKs treated with vehicle or MG132. Right: normalized UBC9 expression. Data are expressed as fold over the untreated empty-transduced HKs. Bars represent means ± SEM of n = 5 different biological replicates. ns: not significant (Kruskal–Wallis one-way ANOVA with Dunn’s post hoc test) compared to vehicle control groups. **(B)** Left: Representative WB of HaCaT cells treated with vehicle or the indicated proteasome inhibitors. Right: normalized UBC9 expression. Data are expressed as fold over untreated cells. Bars represent means ± SEM of n = 6 different biological replicates. ns: not significant (Kruskal–Wallis one-way ANOVA with Dunn’s post hoc test) compared to vehicle control groups.

Next, to shed light on how HPV16 E6/E7 increased UBC9 protein level, we determined the amount of Ubc9 transcript by Real Time-qPCR (Table A in S1 Text). Our results indicate that UBC9 protein accumulation occurred at a post-translational level since Ubc9 mRNA was not affected by HPV16 E6/E7 expression (Fig C in S1 Text). Therefore, we examined the possibility that HPV16 E6/E7 affected UBC9 proteasomal degradation, similarly to what previously reported for the Gam1 viral protein [35] and HPV E6 in a different cellular background [36]. Treatment with the proteasome inhibitor MG132 did not promote UBC9 accumulation in neither empty nor HPV16 E6/E7 HKs (Fig 6A), in contrast to the accumulation of the well-known proteasome substrate in E6 expressing cells, p53 [7]. These results suggested that UBC9 is not physiologically degraded by the ubiquitin-proteasome system (UPS) in HKs. We also analyzed whether UBC9 could be degraded by the UPS in another keratinocyte cell line by treating HaCaT cells (Fig 6B) with three different well-known proteasome inhibitors (MG132, Bortezomib, or Epoxomicin). Even though all tested drugs induced accumulation of ubiquitylated proteins, none of them increased UBC9 levels when compared to vehicle-treated cells, confirming that UBC9 cannot be degraded by UPS in keratinocytes. Thus, these results indicated that HPV16 E6/E7 selectively up-regulate UBC9 expression in HKs affecting UBC9 degradation, but not through the UPS.

**Fig 7:**
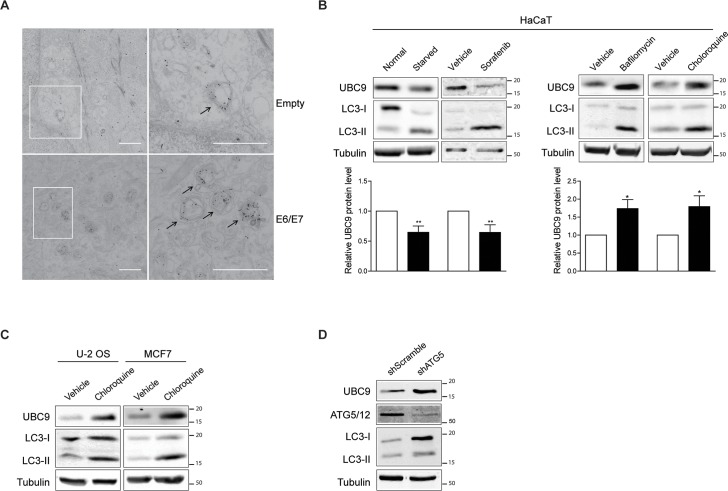
UBC9 is degraded by autophagy in epithelial cells. **(A)** Immunolocalization of UBC9 in ultrathin sections of HKs transduced with empty or HPV16 E6/E7 vectors. Original view (left) and higher magnification (right) of boxed regions. Gold particles are selectively enriched in autophagic structures highlighted by arrows. Scale bar = 1 μm. **(B)** Top: Representative WB of HaCaT cells treated with the indicated autophagic activators (left) and inhibitors (right). Activation of autophagy was monitored by the conversion of LC3 (LC3-I) to the lipidated LC3 (LC3-II) form, a marker of autophagosome production induced by autophagic stimuli [41]. LC3-II accumulation was used to verify autophagic impairment [41]. Bottom: normalized UBC9 expression. Data are expressed as fold over untreated cells. Bars represent means ± SEM of n = 4 different biological replicates. ns: not significant (Kruskal–Wallis one-way ANOVA with Dunn’s post hoc test) compared to vehicle control groups. (**C)** Representative WB analysis of U-2 OS or MCF7 cells treated with chloroquine. n = 3 different biological replicates. **(D)** Representative WB analysis of MCF7 cells transduced with scramble or ATG5 shRNA. The observed ATG5 band represents the ATG5-ATG12 conjugated form. LC3-I accumulation is reported to evidence autophagic deficiencies promoted by shATG5. n = 3 replicates of a single transduction.

We then analyzed the autophagy-lysosomal pathway, the other main cellular route responsible for protein degradation that usually accounts for degradation of long-lived proteins and damaged organelles in lysosomal vacuole (reviewed in [37]). During macroautophagy (hereafter simply referred as autophagy) autophagic cargos are sequestered inside the phagophore, leading to the formation of the autophagosome. Subsequently, the autophagosome fuses with the lysosome giving rise to the autolysosome where sequestered materials are degraded by lysosomal hydrolases. Since an autophagic-degraded protein should be present inside autophagosomes, we firstly investigated through transmission electron microscopy (TEM) whether UBC9 could be enclosed inside the peculiar double-membranes structures which identify autophagosomes [38]. TEM analysis showed the accumulation of UBC9 gold particles in membrane-bound structures resembling autophagosomes (Fig 7A) both in empty and in HPV16 E6/E7 HKs, suggesting an autophagic dependent route for UBC9 degradation. Consistently, we found that stimulation of autophagic degradation by both keeping cells in starvation medium [39], and by treatment with the multi kinase inhibitor sorafenib [40] induced UBC9 degradation (Fig 7B). Similarly, treatment of HaCaT cells with the late autophagy inhibitors chloroquine or bafilomycin A1 [41] resulted in UBC9 accumulation, confirming that UBC9 is targeted by autophagy in keratinocytes. Moreover, UBC9 degradation by autophagy occurred in other cell lines in addition to keratinocytes since chloroquine treatment induced UBC9 accumulation also in U-2 OS and MCF7 (Fig 7C) cells. Finally, UBC9 accumulated in cells in which autophagy was depleted of the essential ATG5 gene [41] (Fig 7D). Therefore, ultrastructural analysis, pharmacological and genetic approaches demonstrated that UBC9 undergoes autophagosomal degradation in diverse epithelial cells.

### HPV16 E6/E7 impair late steps of basal autophagy

**Fig 8:**
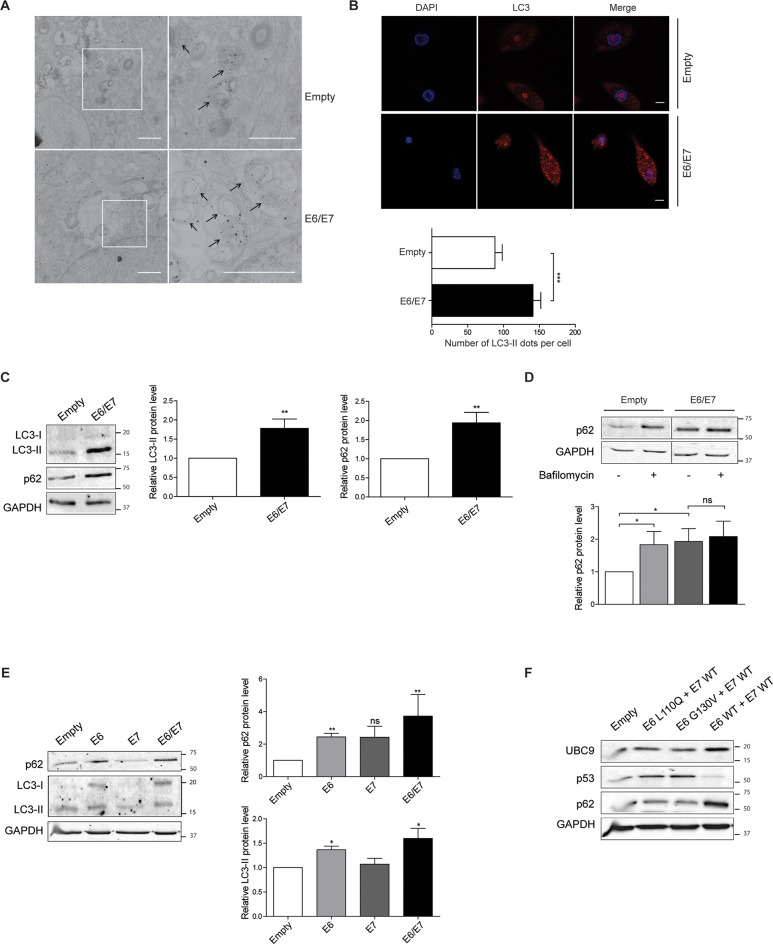
HPV16 E6/E7 inhibit late autophagy. **(A)** Immunolocalization of LC3 in ultrathin sections of HKs transduced with empty or HPV16 E6/E7 vectors. Original view (left) and higher magnification (right) of boxed regions. Nanogold particles are selectively enriched in autophagic structures highlighted by arrows. Scale bar = 1 μm. **(B)** Top: Confocal microscopy images of empty or HPV16 E6/E7 transduced HKs immunostained with antibody against LC3 (red). Nuclei were stained with DAPI (blue). Scale bar = 5 μm. Bottom: Quantification of endogenous LC3 dot total area. Data are expressed as number of LC3 dots. Bars represent means ± SEM of n = 50 fields of three different biological replicates. ***P < 0.0001 (independent-sample t test). **(C)** Left: Representative WB analysis of LC3 and p62 in empty- or HPV16 E6/E7-transduced HKs. Right: LC3 and p62 normalized expression. Data are expressed as fold over empty HK. Bars represent means ± SEM of n = 18 different biological replicates. **P < 0.001 (one-sample t-test). **(D)** Left: Representative WB analysis of empty- or HPV16 E6/E7-transduced cells treated with vehicle or bafilomycin for 8 hours. Right: normalized p62 expression as fold over empty cells. Bars represent means ± SEM of n = 10 different biological replicates. **P < 0.001 (Kruskal–Wallis one-way ANOVA with Dunn’s post hoc test). **(E)** Left: Representative WB analysis of LC3 and p62 in empty-, E6-, E7-, or HPV16 E6/E7-transduced HKs. Right: LC3 and p62 normalized expression. Data are expressed as fold over empty HK. Bars represent means ± SEM of n = 8 different biological replicates. ns: not significant, *P<0.05, **P < 0.001 (Kruskal–Wallis one-way ANOVA with Dunn’s post hoc test) **(F)** Left: Representative WB analysis of UBC9 and p62 expression HKs transduced with the indicated combination of HPV16 E6 wt or mutants and E7 wt. n = 3 biological replicates.

Next we asked whether UBC9 increase in HPV16 E6/E7 cells could be due to autophagy alteration. Therefore, we monitored autophagosome formation carrying out TEM experiments upon LC3 immunogold labeling. LC3 could exist as diffuse cytosolic LC3-I or as autophagosomes-loaded LC3-II after lipidation with phosphatidylethanolamine. Evaluation of LC3-II dots is thus a measurement of autophagosomes number and allows autophagy monitoring [41]. In HPV16 E6/E7, as compared to empty HKs (Fig 8A), TEM analysis showed a marked enrichment in autophagosome-like LC3 positive membrane-bound structures [38], suggesting autophagy alteration promoted by HPV16 E6/E7. Notably, the same LC3 positive structures morphologically resembled the autophagic structures that also contain UBC9 (Fig 7A), further indicating the involvement of the autophagic-degradation pathway in UBC9 clearance. We then quantified LC3 dots in HKs transduced with empty or HPV16 E6/E7 retroviral vectors by means of confocal microscopy analysis. Assessment of endogenous LC3 revealed an increased number of LC3-II dots in HPV16 E6/E7 HKs (Fig 8B), consistently confirmed by WB analysis of lipidated LC3 (Fig 8C). Increased autophagosomes number could be an effect of induced autophagosome formation or as the consequence of suppression in late autophagic steps [41]. Thus, we assessed expression of the specific autophagic substrate p62, whose total levels inversely correlate with autophagic activity [42]. Similarly to LC3-II, we observed that also p62 accumulated in HPV16 E6/E7 compared to empty HKs (Fig 8C), suggesting that HPV16 E6/E7 expression leads to late defects in autophagic degradation rather than in promoting autophagy. LC3 and p62 were not transcriptionally regulated by HPV16 E6/E7 since their mRNAs expression was not affected by HPV16 E6/E7 (Fig D and Table A in S1 Text), confirming that their increase was due to autophagic defects. We then evaluated autophagic flux by LC3 turnover assay [43]. Failure of LC3-II accumulation in bafilomycin A1-treated cells would indicate an early autophagic defect before lysosome degradation [43]. However, treatment with the late autophagic inhibitor bafilomycin A1 [41] efficiently accumulated LC3-II, indicating efficient autophagosome formation in both empty and HPV16 E6/E7 HKs (Fig E (a) in S1 Text), and thus excluding early autophagy deficiencies prompted by HPV16 E6/E7. Notably, in the same experimental conditions, we detected significant p62 accumulation in empty but not in HPV16 E6/E7 HKs (Fig 8D) bafilomycin A1-treated cells, suggesting that despite normal autophagosome formation the expression of the viral oncoproteins directly affected lysosomal degradation capability. Additionally, we also found that the impairment in later steps of autophagy was mainly due to E6 since its expression alone was able to increase both LC3-II and p62 levels in infected HKs, although further enhanced by E7 (Fig 8E) as previously reported [44]. Finally, since our results highlight a p53-dependent mechanism mediated by E6 to enhance UBC9 expression (Fig 5), we monitored autophagy status in HKs co-transduced with the E6 mutants defective for p53 degradation (L110Q and G130V) or E6 WT, respectively, and E7 WT. In agreement with our previous results (Fig 5), we observed that both the HPV16 E6 mutants were less efficient than the E6 WT in cooperating with E7 to promote UBC9 accumulation in HKs (Fig 8F). Similarly, E6 WT was less effective in triggering UBC9 overexpression when combined with E7 C24G as compared to E7 WT (Fig F in S1 Text). Moreover, consistently with our hypothesis, L110Q and G130V mutants (whose expression was verified by RT-qPCR, Fig G in S1 Text) dampen the ability of E6 WT to increase p62 expression in combination with E7, suggesting a functional autophagic flux in absence of p53 degradation. Hence, our data showed that HPV16 E6/E7 impaired late stages of autophagy mainly through an E6/p53-dependent mechanism.

### HPV16 E6/E7 reduce autophagosome-lysosome fusion in HKs

Suppression in late autophagic steps could arise from impared lysosomal proteolytic activity or from deficient autophagosome-lysosome fusion [41]. To distinguish between these two possibilities, we monitored lysosomal proteolytic functionality with the EGFR (epidermal growth factor receptor) degradation assay, where the rate of degraded EGFR is a direct measure of digestion of endosomal cargo by lysosomes [45]. In this assay we found that EGFR degradation was similar in empty and HPV16 E6/E7 HKs (Fig E (b) in S1 Text), ruling out lysosomal breakdown defects prompted by HPV oncoproteins.

**Fig 9:**
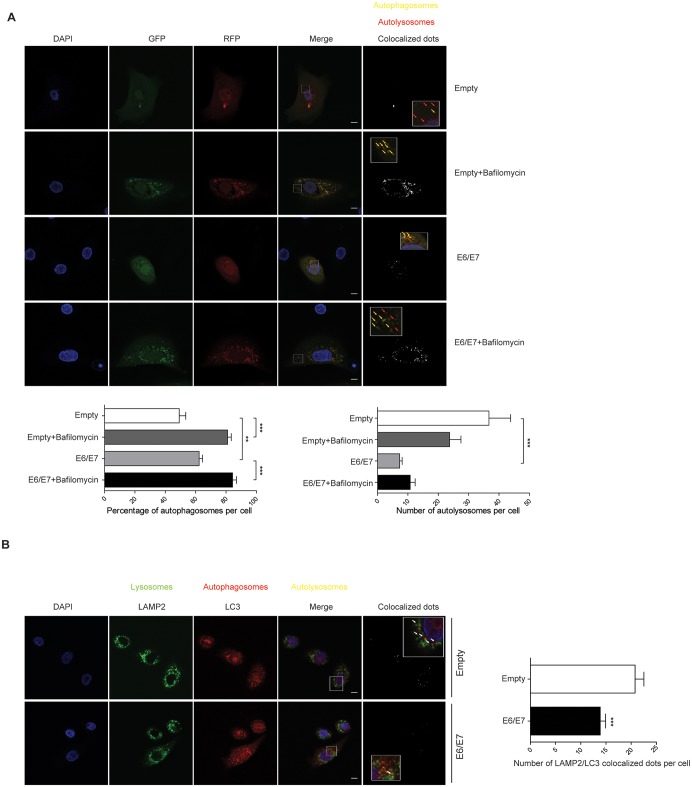
HPV16 E6/E7 impair autolysosomes formation. **(A)** Top: Confocal microscopy images of empty or HPV16 E6/E7 HKs expressing the GFP-LC3-RFP construct and treated with bafilomycin A1 for 8 hours. Nuclei were stained with DAPI (blue). Colocalized dots images show GFP/RFP dots colocalization as determined by the Green and Red Puncta Colocalization plugin of ImageJ. Inset show higher magnification views of boxed regions. Yellow arrows highlight colocalized dots (autophagosomes), red arrows indicate autolysosomes. Scale bar = 5 μm. Bottom: quantitation of percentage of yellow puncta (autophagosomes) or red only puncta/per cell (autolysosomes). Data are expressed as percentage of yellow dots or as number of only red dots, respectively, as measured by the Green and Red Puncta Colocalization plugin. Bars represent means ± SEM of n = 45 fields of three different replicates. **P < 0.001; ***P < 0.0001(one-way ANOVA followed by Bonferroni post hoc test). **(B)** Left: Confocal microscopy images of empty or HPV16 E6/E7 HKs immunostained with antibodies against LAMP2 (green) and LC3 (red). Nuclei were stained with DAPI (blue). Colocalized dots images show LAMP2/LC3 dots colocalization as determined by the Green and Red Puncta Colocalization plugin. Inset show higher magnification views of boxed regions. Scale bar = 5 μm. Right: Quantification of LAMP2/LC3 colocalized dots per cell. Data are expressed as number of LAMP2 dots colocalized with LC3 dots as measured by the Green and Red Puncta Colocalization plugin. Bars represent means ± SEM of n = 95 fields of five different biological replicates. ***P < 0.0001 (independent-sample t test).

Hence, we explored the possibility that the late autophagic deficiencies may be ascribed to a reduced fusion between autophagosomes and lysosomes, a key event for autolysosome formation, lysosomal activation and degradation of autophagic cargo [46]. To this end, we computationally counted the number of autophagosome and autolysosome structures in control or HPV16 E6/E7 HKs transduced with a RFP-GFP-LC3 construct [47]. Since GFP loses its fluorescence in the acidic pH, autolysosomes (pH about 4.5–5) are shown by only RFP signals, while autophagosomes are shown by both GFP and RFP as yellow puncta. Using this plasmid and confocal miroscopy, we showed that the percentages of autophagosomes were significantly increased in HPV16 E6/E7 HKs, validating our earlier results (Fig 8A). Markedly, HPV16 E6/E7 HKs also contained very few autolysosomes (Fig 9A), suggestive of fusion defects between autophagosomes and lysosomes. In addition, bafilomycin A1 treatment failed to further decrease autolysosomes number in HPV16 E6/E7 cells, indicating that HPV16 E6/E7 mimics bafilomycin effects in HKs. Therefore, these data revealed a decreased autolysosome formation in HPV16 E6/E7 HK, suggesting that the fusion between autophagic vacuoles and lysosomes was inhibited. To confirm this observation, we then imaged by confocal microscopy empty or HPV16 E6/E7 HKs stained for endogenous LC3 as marker of autophagosomes, and endogenous LAMP2 as a marker of lysosomes. Our computational quantification of autolysosomes number (LC3-LAMP2 positive dots) again showed that HPV16 E6/E7 HKs are characterized by a smaller extent of colocalized dots as compared to empty HKs (Fig 9B), once more pointing to autophagosome-lysosome fusion defects triggered by HPV16 E6/E7.

### UBC9 accumulates in HPV16 E6/E7 HKs and cervical lesions due to autophagic defects

**Fig 10:**
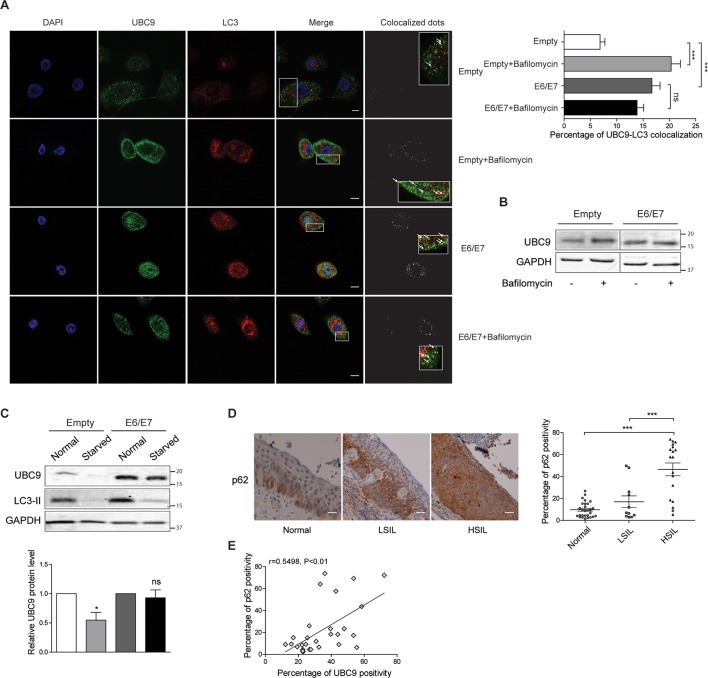
Late autophagic defects account for UBC9 accumulation in HPV16 E6/E7 HKs and cervical lesions. **(A)** Left: Confocal microscopy images of empty or HPV16 E6/E7 transduced HKs treated with vehicle or bafilomycin for 24 hours, and immunostained with antibody against UBC9 (green) and LC3 (red). Nuclei were stained with DAPI (blue). Colocalized dots images show UBC9/LC3 dots colocalization as determined by the Green and Red Puncta Colocalization plugin. Inset show higher magnification views of boxed regions. Arrows highlight colocalized dots. Scale bar = 5 μm. Right: Quantification of UBC9/LC3 colocalization expressed as percentage of colocalized UBC9 with LC3 (double labeled dots) dots on total UBC9 (single labeled plus double labeled dots) per cell, as determined with the Green and Red Puncta Colocalization plugin. Bars represent means ± SEM from at least 47 fields of three different biological replicates. ***P < 0.0001; ns: not significant (one-way ANOVA and Bonferroni post hoc test). **(B)** Representative WB analysis of UBC9 expression in cells treated as in (A). n = 3 different biological replicates. **(C)** Top: Representative WB analysis of empty or HPV16 E6/E7 HKs cultured in growth or in starvation medium. LC3-II disappearance was used to confirm autophagy activation [92]. Bottom: UBC9 normalized expression reported as fold over cells grown in normal medium. Bars represent means ± SEM of n = 7 different biological replicates. *P<0.05; ns: not significant (Kruskal–Wallis one-way ANOVA with Dunn’s post hoc test) compared to normal medium-grown cells. **(D)** Left: Representative IHC images of FFPE human normal, LSIL or HSIL cervical tissues stained with anti-p62 antibody. Right: Percentage of p62 positive pixels in FFPE human cervical tissues. Scale bars = 200nm. Data are expressed as percentage of positive plus strong positive stained pixels. The black lines represent the mean for each group. n = 18 (normal), 11 (LSIL), 18 (HSIL) different patients. ***P < 0.0001 (one-way ANOVA and Bonferroni post hoc test). **(E)** Correlation between p62 and UBC9 expression human cervical tissues. n = 29 different patients. Scattered plots with linear regression line, Spearman r correlation coefficient, and p value are reported.

We then next asked whether autophagic defects might explain the higher UBC9 protein level observed in HPV16 E6/E7 expressing cells and tissues. Consistently with our TEM data (Figs 7A and 8A), we found by confocal microscopy that UBC9 and LC3 truly colocalized in empty HKs, and that the extent of colocalization was significantly increased by treatment of cells with bafilomycin A1, supporting an autophagic degradation pathway for UBC9 (Fig 10A). In contrast, the rate of UBC9 dots colocalized with LC3-II were basally higher in HPV16 E6/E7 cells as compared to empty HKs and were completely unaffected by bafilomycin A1 (Fig 10A). Similar results were also obtained by WB, in the same experimental conditions (Fig 10B). Therefore, HPV16 E6/E7 impaired UBC9 autophagosomal degradation and promoted UBC9 accumulation by inhibition of later stages of autophagy, phenocopying bafilomycin A1 effects. Correspondingly, autophagy activation by nutrient deprivation [39] significantly promoted UBC9 degradation only in empty HKs implying autophagic defects in HPV16 E6/E7 cells that finally impaired UBC9 clearance (Fig 10C). In addition, to understand whether autophagy impairment also occurred *ex vivo*, we monitored p62 accumulation as a readout of autophagic deficiencies [42] during lesion progression in FFPE sections of normal, LSIL or HSIL tissues. Qualitative and quantitative IHC analysis (Fig 10D) showed that p62 expression was increased during lesion progression, indicating autophagic defects. Moreover, correlation analysis of p62 and UBC9 positivity in matched pairs of FFPE cervical tissues showed a positive correlation of UBC9 with p62 (Fig 10E), suggesting a strict association between UBC9 expression and p62 levels. Therefore, these results illustrated that autophagy is impaired during cervical lesion progression, and that UBC9 expression in the human cervix is closely linked to a functional autophagic degradation pathway.

## Discussion

**Fig 11:**
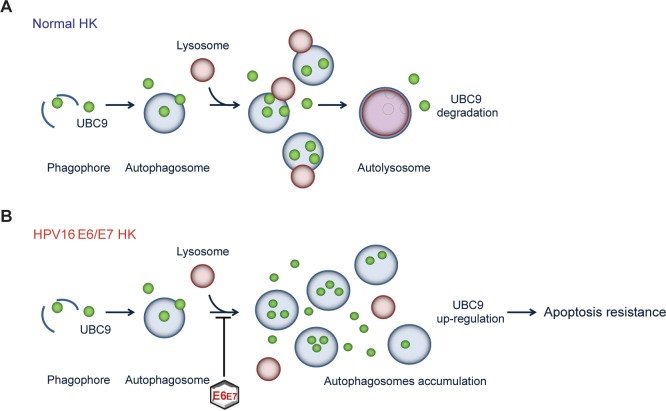
Model of autophagic-mediated UBC9 degradation and its impairment by HPV16 E6/E7 in HKs. **(A)** In normal conditions, UBC9 protein expression is retained at physiological levels by basal autophagy in HKs. **(B)** However, the presence of HPV16 E6/E7 impairs autophagosome-autolysosome fusion. As a consequence, UBC9 is no longer efficiently cleared by the autophagic machinery and accumulates in HPV16 E6/E7 transduced cells. Finally, the increased UBC9 expression leads to extended life-span of HPV16 E6/E7 HKs.

In this study we unveil how HPV E6/E7 oncoproteins manipulate the SUMO pathway through a selective up-regulation of the SUMO conjugating enzyme UBC9. Indeed, we show that UBC9 is physiologically degraded by the autophagic-lysosome pathway. However, the presence of HPV16 E6/E7 oncoproteins impaired autophagosome-lysosome fusion leading to UBC9 overexpression which contributes to protect HKs from apoptotic cell death (Fig 11). The extended lifespan of HPV-transduced cells sustained by UBC9 may therefore increase viral persistence in the host, as demonstrated in other viral systems [18–20], possibly contributing to promote lesion progression and cancer growth. Indeed, although oncoviral infection is a primary cause of tumorigenesis, only a minor percentage of infected individuals develop invasive growth, indicating that viral oncogenes expression is not sufficient and other events are needed for cancer to occur [1]. We thus propose that endogenous UBC9 status may be another important cofactor that, by delaying apoptotic clearance of transduced cells, ensures viral persistence in the host.

Since SUMOylation is a highly reversible PTM able to control almost all cellular activities such as transcriptional regulation, DNA repair, cell growth and apoptosis [9], a variety of viruses have developed clever strategies to manipulate SUMO to their own advantage [18–20]. Consistently, HPV exploit the SUMO pathway through early [36,48–51] and late [52,53] proteins to support different steps of its infection cycle. It has been shown that E6 is capable of reducing UBC9 levels in several cell lines [36], apparently in contrast with our findings. However, UBC9 overexpression documented here (Fig 2A) was obtained mimicking HPV16 E6/E7 expression in its natural host, primary human keratinocytes, and we also previously provided evidence of UBC9 up-regulation in human cervical tissues [26] also confirmed in the present work (Fig 1A). We therefore speculate that the specific cellular background might be a critical determinant that drives UBC9 fate. In support of this hypothesis, we provided evidence demonstrating that UBC9 upregulation is driven by HPV16 E6/E7 also in another primary cell, namely human fibroblasts (Fig 5B), and is intimately connected to the p53 status of transduced cells (Fig 5). Therefore, a possible mechanistic explanation of UBC9 output after HPV16 E6 expression may reside in the p53 functionality of studied cells: overexpressed in primary (that harbor normal p53), degraded in immortalized (with usually altered p53). In addition, both LR and HR HPV E6/E7s, and SV40 large T antigen up-regulate UBC9 expression (Fig 4A and 4B) implying an activity of conserved relevance for the different virus life cycles, and p53 activity as well [54,55], further validating our hypothesis that poses UBC9 overexpression as a key feature during viral infections. Indeed, by targeting a single molecule extensively involved in physiological activity, viral proteins may completely subvert cells behavior to virus advantage. In agreement with this hypothesis, we showed that UBC9 expression finely regulates HKs apoptosis (Fig 4C and 4D) similarly to what previously found in other systems [22,25,56]. Indeed, the essential SUMO conjugating enzyme UBC9 is a central protein for many cellular activities and could also be a fundamental driver of cancer growth since it is altered in a variety of human malignancies [21], and thus a putative candidate for drug therapy [57]. Our results strengthen this view and extend the potential use of targeting UBC9 also to induce death in HPV-transduced cells (Fig 4C), usually very resistant to apoptosis [31], in order to facilitate their clearance. In addition to the therapeutic implications, our findings also indicate that UBC9 could be a useful biomarker not only for cervical progression [26], but also to monitor and to identify pre-cancerous stages in HNC (Fig 1), extending previous observation made in head and neck tumor tissues [58]. Importantly, our IHC results (Fig 1B and 1C) link higher UBC9 expression with HPV presence, suggesting the potential use of UBC9 as a surrogate marker to specifically distinguish HPV-positive from HPV-negative infections in HNC. Patients with HPV-positive tumors are usually younger and have a disease that is more responsive to therapy and an overall better prognosis [59]. Therefore, the definition of UBC9 as a new predictor to help in detecting more easily HPV-positive pre-cancer tissues could represent a significative advancement for the accurate clinical management of HNC patients.

Our work also sheds light on how levels of an important enzyme in tumorigenesis, UBC9, are physiologically regulated. Indeed, we provide ultrastructural, pharmacological, and genetic evidence supporting the notion that UBC9 is degraded by autophagy in several epithelial cells (Fig 7). Moreover, the recent findings that UBC9 itself may regulate autophagy [60] could open an intriguing scenario where autophagy finely tunes levels of one of its regulator through a feed-forward loop. UBC9 degradation through autophagy is also in agreement with recent findings in which an inhibitor of lysosomal enzymes [61], but not proteasomal inhibitors [36,61], rescued pathogens-induced UBC9 degradation. Opposite, we previously found that another viral protein, Gam1, stimulated proteasomal-dependent UBC9 degradation [35], raising the possibility that different cellular stresses and pathological conditions might differently alter UBC9 clearance, in order to finely tune its protein levels either through the UPS or autophagy.

Our results also depict a peculiar pattern of UBC9 and SUMO expression during cervical lesion progression, pointing to a specific role for UBC9 in driving SUMO paralog specificity (Figs 2 and 3) promoted by E6/E7. In line with recent evidence showing an important function for UBC9 PTMs or for the oxidative status in determining SUMO substrate selectivity [62–66], it is possible that HPV16 E6/E7 not only affect UBC9 expression, but could also selectively regulate its enzymatic activity by modifying its PTM code during lesion progression. Moreover, although details of SUMO2/3 conjugation in response to cellular stresses such as viral infection are quite well understood [67,68] little is known about mechanisms and functions of selective SUMO1 up-regulation and its role during cellular transformation. Therefore, our results could possibly open important pathobiological implications to further investigate.

Finally, we uncover how HPV16 E6/E7 alter autophagy. HPV16 early genes negatively impact autophagy [69], and in particular the contribution of HPV16 E5 protein has been recently described [70]. Additionally, inhibition of host autophagy significantly enhanced HPV infectivity and its tumorigenic potential [71–74], further underlining the notion that HPV turns off autophagy through many different mechanisms to promote its infection cycle. Here we further support these data showing that the main HPV oncoproteins E6/E7, mostly owing to E6 action on p53, contribute to the autophagic pathway impairment during cellular transformation, by selective inhibition of autolysosome formation (Figs 8 and 9 and Fig E in S1 Text). In support of our results, it has been recently shown that in anuses of HPV K14E6/E7 transgenic mice and in anal human samples there was an increase in LC3, p62, and autophagosomes structure without evidence of autolysosomes, completely confirming our findings also in a different kind of HPV-derived malignancy [75]. p53 roles in autophagy regulation are as complex as fascinating: indeed, p53 has been reported to act both as positive or negative guardian of autophagic pathway, depending on its cellular localization [76–80]. Despite mechanisms of autophagy activation by p53 are quite well understood, little is known about its function as autophagic repressor [76–81]. Therefore, more efforts are needed to better understand how the E6/p53 axis contributes to the autophagic impairment revealed by our studies. It is also unknown whether E7 triggers UBC9 up-regulation (Fig 5A) through autophagy or through different mechanisms, and future works are surely needed to better clarify this issue. Furthermore, we showed that autophagy could be clinically relevant to carcinogenesis, since this process is also impaired during lesion progression (Fig 10D), underlining that also HPV hijacks autophagy to promote malignant transformation as several other viruses [82,83]. Consistently, the recent findings that p62 accumulates in HNC tissues also confirmed that autophagy is relevant in HN tumorigenesis [84]. Therefore, it is conceivable to propose autophagy-stimulating strategies to control the progression of early stages of both cervical and HN carcinogenesis. Moreover, in addition to promoting UBC9 up-regulation (Fig 10), autophagic impairment could also lead to several other consequences (i.e. p62 accumulation) whose elucidation would certainly help to better characterize the HPV-induced diseases.

In conclusion, our findings candidate UBC9 and autophagy as novel players of viral carcinogenesis, and open innovative possibilities for attractive therapeutic strategies against tumor progression in HPV-related malignancies.

## Materials and methods

### Cells

Phoenix Ampho and 293T (ATCC), HaCaT, U-2 OS, MCF7, HeLa (CLS), and primary human fibroblasts were maintained in DMEM plus 10% fetal bovine serum (FBS, Cambrex). Caski (ATCC) were maintained in RPMI plus 10% FBS. Cells were routinely authenticated with GenePrint 10 System (Promega). Adult human epidermal keratinocytes (HKs) were prepared and maintained as previously described [85]. Human primary dermal fibroblasts (HFs) were isolated from skin punch biopsies, cut to approximately 1 cm^2^ pieces and digested with Dispase (10 U/mL; Gibco) for 4 h at 37°C to separate epidermis from derma. Excised derma was put in 6-well plates, filled with DMEM+FBS for 2 weeks changing medium every other day until obtainment of primary fibroblasts colonies. HKs and HFs from passages 1 to 5 were used for the experiments. All cells were maintained at 37°C with 5% CO_2_.

### Transductions, transfections, and plasmids

For retroviral transduction, plasmids were transfected into Phoenix Ampho cells by calcium-phosphate method. The following day, cells were transduced with retroviral supernatants for 6 h at 37°C for two days and selected with G-418 Sulfate (Gibco) for 1 week and finally collected for RNA extraction, WB or treated with drugs. pLKO shScramble/shATG5, and pLenti CMV GFP-LC3-RFP plasmids were transfected in 293T cells by calcium-phosphate method. The following day, HKs were transduced with lentiviral supernatants for 6 h at 37°C for two days and selected with puromycin (Sigma Aldrich) for 3 days, and collected WB or fixed for imaging experiments. For apoptosis evaluation, HaCaT cells were transfected with pcDNA3 empty or pCDNA3 UBC9 plasmid using Lipofectamine 2000 (Thermo Fisher Scientific) following manufacturers’ instruction. 48 hours after transfection, apoptosis was stimulated as described below. For evaluation of UBC9 expression after E6/E7 depletion, shScramble and shp53 plasmids were transfected into Phoenix Ampho cells by calcium-phosphate method. The following day, Caski were transduced with retroviral supernatants for 6 h at 37°C for two days and selected with puromycin (Gibco) for 1 week. Selected cells were then seeded on 6well plates and transfected with Lipofectamine 2000 (Thermo Scientific) with siLuc or two different E6/E7 siRNAs following manufacturers’ instruction. 48 hours after transfection, cells were collected for WB analysis.

pLXSN-6 E6/E7 was generated by subcloning inserts from pBABE puro-6 E6/E7 (kind gifts by D.J. McCance, Queen’s University Belfast, Belfast). pZIP SV40 large T antigen, pBABE shp53, and pBABE shRb were a gift from D. Pasini (European Institute of Oncology, Milan). pLKO puro shScramble and shATG5 were kindly provided by S. Minucci (European Institute of Oncology, Milan). pLenti CMV GFP-LC3-RFP was a generous gift from J. Yue (University of Hong Kong, Hong Kong, China).

### RNA extraction, reverse transcription and qRT-PCR

Total RNA was extracted with the RNeasy mini kit (Qiagen), following the manufacturer’s instruction, and reversely transcribed using the ImProm-II system for RT-PCR (Life Technologies). RT-qPCR was performed with the ABI Prism 7900 Sequence Detection System, using the Fast SYBR Green Master Mix (Applied Biosystems) and the specific primer pairs listed in Table A in S1 Text. The expression of the indicated mRNAs was quantitated by the comparative ΔΔCt method. GAPDH was used as control for normalization.

### Immunoblotting and antibodies

Cells were lysed in RIPA buffer plus protease inhibitors (leupeptin 1 μg/mL, aprotinin 1 μg/mL, PMSF 100 μg/mL and EDTA 1 mM) and 0.5 mM N-ethylmaleimide (NEM). Equal amount of total proteins were resolved by SDS-PAGE under reducing conditions, and immunoblotted with the indicated antibodies: anti-UBC9 (Abcam), anti-SUMO1 (Sigma-Aldrich), anti-SUMO2/3 (Abcam), anti-RanGAP1 (Santa Cruz Biotechnology), anti-p53 (DO-1; Santa Cruz Biotechnology), anti-pRb (Santa Cruz Biotechology), anti-SAE1 (Abcam), anti-SAE2 (Abcam), anti-Ubiquitylated proteins (FK1, Biomol), anti-LC3 (Novus Biologicals), anti-p62 (2C11, Abnova), anti-EGFR (kindly provided by S. Sigismund, Fondazione Istituto FIRC di Oncologia Molecolare, IFOM, Milan [86]), anti-ATG5 (Cell Signaling Technology), anti-pRB (BD Pharmingen). Anti-GAPDH (Abcam), anti-tubulin (Sigma-Aldrich), or anti-vinculin (Santa Cruz Biotechnology) antibodies were used as loading control. Membranes were then incubated with the appropriate IR Dye-conjugated or horseradish peroxidase (HRP) secondary antibodies (Licor), and scanned with a LI-COR Odyssey Imager or acquired with Chemidoc (Bio-rad), respectively. The intensities of the protein bands were quantified using ImageJ software (Rasband W.S., ImageJ, U. S. National Institutes of Health), and standardized to the housekeeper levels.

### RNA interference

Control small interfering RNA (siRNA against luciferase gene, siLuc) or two different Ubc9 siRNAs were transfected with RNAiMAX (Life Technologies), following manufacturer instructions. Cells were harvested 72 hours after transfection and immediately processed for flow cytometry. siRNAs sequences were listed in Table B in S1 Text. Short hairpin RNA (shRNA) against mock sequence (shScramble) or ATG5 (shATG5) were transduced in MCF7 cells as previously described. shRNA sequences were previously reported [87].

### Apoptosis assay and flow cytometry

For apoptosis evaluation, 5x10^5^ cells were incubated for 15 minutes at room temperature with Annexin V-FITC (Sigma-Aldrich), washed, incubated with 50 μg/ml Propidium Iodide (PI) and immediately processed. Samples were acquired using a FACSCalibur flow cytometer (Becton Dickinson), and data were analyzed using CellQuest software (Becton Dickinson), reporting early (Annexin V^+^PI^-^) and late (Annexin V^+^PI^+^) apoptotic cells. UV stimulation was performed with UV Stratalinker (Stratagene), exposing HaCaT cells with 30J/m^2^ ultraviolet light. 24 hours after UV stimulus cells were stained with Annexin V-FITC/PI and analyzed for flow cytometry as described above.

### Patients and HPV testing

For cervical specimens, patients were enrolled at European Institute of Oncology form 2001 to 2013. Patients characteristics were previously reported [26]. HPV DNA and RNA testing was performed as previously reported [88].

### Immunohistochemisty and quantitative analysis of histochemical staining

IHC was performed as previously described [26]. For IHC co-stain, FFPE samples were deparaffinized, antigens retrieved at 99°C for 40 minutes, incubated with the first stain antibody (UBC9) O/N at 4°C, and visualized using the 3,3’-diaminobenzidine (DAB) chromogen. Then, samples were treated with Denaturing Solution (Biocare) to avoid cross-reaction, stained with the second antibody (SUMO1), and visualized with LSAB-AP and Vulcan Fast Red. Finally, slides were counterstained with haematoxylin and coverslipped. The following primary antibodies were used: anti-UBC9 (H-81, Santa Cruz Biotechnology), anti-SUMO1 (Santa Cruz Biotechnology), anti-SUMO2/3 (Abcam), anti-p62 (2C11, Abnova), anti-Ki-67(MIB-1, Dako). Images were generated with a BX51 Upright Microscopes from Olympus America Inc at 20x magnification. For morphological pathologist assessment, the Low Grade or High Grade dysplasia were obtained using morphological grading system and the percentage of positive staining in the tumor cells was quantified by blinded reading of the slide. Computational analysis was carried out as previously described [26].

### Drugs and treatments

HKs were treated with 10 μM MG132 (Sigma Aldrich) for 6 hours, 10 nM bafilomycin A1 (Sigma Aldrich) for 8 or 24 hours as indicated in figure legends, in starvation medium (140 mM NaCl, 1 mM MgCl_2_, 5 mM glucose, and 20 mM Hepes, pH 7.4, modified from [39]) for 4 hours. HaCaT cells were treated with 10 μM MG132 (Sigma Aldrich), 100 nM bortezomib (Aurogene), or 10 μM epoxomicin (Clinisciences), for 8 hours, starvation medium for 4 hours, 10 μM sorafenib tosylate (Aurogene) for 16 hours in medium without FBS, 10 nM bafilomycin A1 (Sigma Aldrich) for 8 hours, or 10 μM chloroquine (Sigma-Aldrich) for 4 hours. U-2 OS and MCF7 were treated with 10 μM chloroquine for 2 hours. Experimental conditions for all treatments were previously determined in preliminary time-course and dose-response experiments.

### Confocal microscopy and quantitative analysis

Cells plated on glass coverslips were fixed with 2% paraformaldehyde, permeabilized with ice-cold methanol for 2 minutes at -20°C, washed and blocked with 5% BSA for 30 minutes. Cells were then incubated with primary antibodies for 45 minutes at room temperature. The following antibodies were used: anti-LC3 (Novus Biologicals), anti-UBC9 (BD Biosciences), anti-LAMP2 (Santa Cruz Biotechnology). Next, cells were incubated with Alexa Fluor (Life technologies) 488 or 647 fluorescent dye-conjugated secondary antibodies for 30 minutes at room temperature. Finally, nuclei were counterstained with DAPI for 5 minutes at room temperature, mounted with high-grade glycerol, and analyzed by confocal microscopy. Empty and HPV16 E6/E7 GFP-LC3-RFP cells were fixed with 2% paraformaldehyde, counterstained with DAPI for 5 minutes at room temperature, mounted with high-grade glycerol, and analyzed by confocal microscopy. Images were captured with a Leica TCS SP2 (Leica Microsystems) using 63X oil objective and acquired with Leica Confocal Software. Quantification of dots number and colocalization were measured using the Red and Green Puncta Colocalization plugin of ImageJ software as previously reported [89].

### Immunogold electron microscopy

Cells were fixed in 4% paraformaldehyde, washed, permeabilized for 10 minutes with 0.25% saponin, 0.1% BSA in PBS, and blocked (0.2% BSA, 5% Normal Goat Serum, 50mM NH_4_Cl, 0.1% saponin, 20mM phosphate buffer, 150mM NaCl). Coverlips were then incubated with primary antibody (anti-UBC9 BD Biosciences or anti-LC3 Novus Biologicals) for 2 hours, washed (0.1% BSA, 0.1% Saponin in PBS) and incubated for 1 hour with secondary antibodies conjugated with nanogold (Nanoprobes). Samples were then fixed with 1% glutaraldehyde for 1 hour and nanogold was enlarged with a gold enhancement solution (Nanoprobes) according to manufacture instructions, postfixed (1% OsO_4_, 1.5% potassium ferrocyanide in 0.1M cacodylate buffer pH 7.4), enbloc stained with 1% uranyl acetate over/night at 4°C, dehydrated with ethanol, embedded in EPON 812 and cured in an oven at 60°C for 48 hours. Ultrathin sections (70-90nm) were cut on an ultramicrotome (Leica FC7, Leica microsystem). Grids were stained with uranyl acetate and Sato’s lead solutions and observed in a Leo 912AB Zeiss Transmission Electron Microscope (Carl Zeiss). Digital micrographs were taken with a 2Kx2K bottom mounted slow-scan Proscan camera (ProScan, Lagerlechfeld, Germany) controlled by the EsivisionPro 3.2 software (Soft Imaging System).

### EGFR degradation assay

EGFR degradation was measured as previously reported [90]. Cells were nutrient-starved (w/o EGF and bovine pituitary extract, BPE) for 4 hours, pretreated with 50 μg/ml cycloheximide (Sigma-Aldrich) for 1 hour, and incubated with 100 ng/ml EGF (Gibco) plus CHX for 30 minutes at 4°C and subsequently shifted for 15 minutes at 37°C. Then, cells were washed and incubated in nutrient-free medium for the indicated time points in the presence of CHX, harvested, and analyzed by WB.

### Statistical analysis

Statistical significance was evaluated with Graphpad Prism version 5.00 software. Multiple samples were compared with ANOVA. Paired or unpaired Student’s t tests were used to compare two samples normally distributed. One-sample t-test was used to compare two non-parametric populations. Spearman rank correlation coefficient was used to test association between variables. Bar represents means ± SEM of the indicated number of biological replicates. Values of P<0.05 were considered significant. *P<0.05; **P < 0.001; ***P<0.0001. ns: not significant.

### Ethics statement

HKs and HFs were isolated from skin biopsies collected via standardized operative procedures approved by European Institute of Oncology Ethical Board. HNC tissues (diagnosed as Oropharyngeal Squamous Cell Carcinoma) were collected through the HPV-AHEAD Consortium. Informed consent was obtained from all cervical and HNC patients.

## Supporting information

S1 Text Supplementary Figure A. Relative mRNAs expression levels of viral proteins in HKs. Supplementary Figure B. HPV E6/E7s from diverse HPV types preferentially promote SUMO1 conjugation *in vitro*. Supplementary Figure C. UBC9 overexpression by HPV16 E6/E7 occurs at post-translational level. Supplementary Figure D. HPV16 E6/E7 do not affect LC3 and p62 transcription. Supplementary Figure E. Lysosomal degradation is not affected by HPV16 E6/E7. Supplementary Figure F. E7 pRb binding mutant partially reduced E6 WT ability to promote UBC9 up-regulation. Supplementary Figure G. Relative mRNAs expression levels of E6/E7 mutants in HKs. Supplementary Table A. Primers used in this study. Supplementary Table B. siRNAs sequences.
(DOCX)
