# Supplementary material for: Autophagy regulates UBC9 levels during viral-mediated tumorigenesis
Source: PLoS Pathog. 2017 Mar 2;13(3):e1006262. doi: 10.1371/journal.ppat.1006262 (PMC5349695; doi:10.1371/journal.ppat.1006262)
Supplement: S1 Text — Supplementary Figure A. Relative mRNAs expression levels of viral proteins in HKs. Supplementary Figure B. HPV E6/E7s from diverse HPV types preferentially promote SUMO1 conjugation in vitro. Supplementary Figure C. UBC9 overexpression by HPV16 E6/E7 occurs at post-translational level. Supplementary Figure D. HPV16 E6/E7 do not affect LC3 and p62 transcription. Supplementary Figure E. Lysosomal degradation is not affected by HPV16 E6/E7. Supplementary Figure F. E7 pRb binding mutant partially reduced E6 WT ability to promote UBC9 up-regulation. Supplementary Figure G. Relative mRNAs expression levels of E6/E7 mutants in HKs. Supplementary Table A. Primers used in this study. Supplementary Table B. siRNAs sequences. (DOCX) [file ppat.1006262.s001.docx]

**S1 Text**


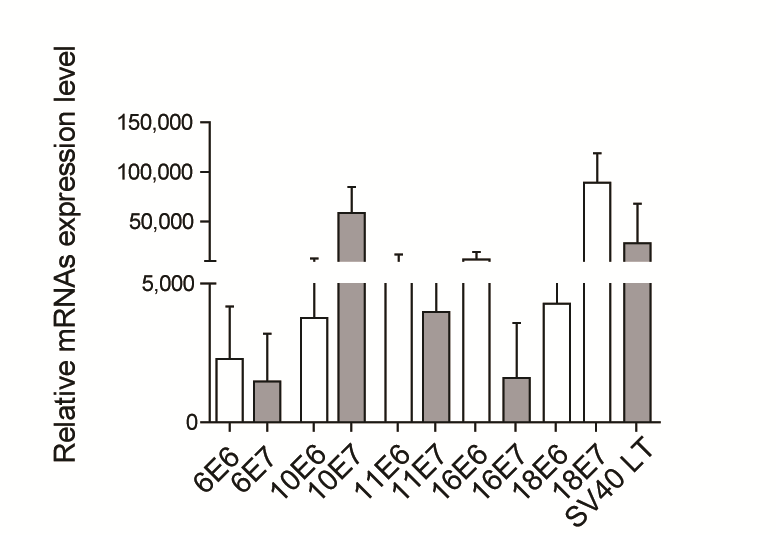


**Figure A. Relative mRNAs expression levels of viral proteins in HKs.** RT-qPCR analysis of viral proteins expression in HKs using specific primers listed in Supplementary Table 1. Data are expressed as fold over the empty-transduced HKs. Bars represent means ± SEM of at least n=3 different donors.


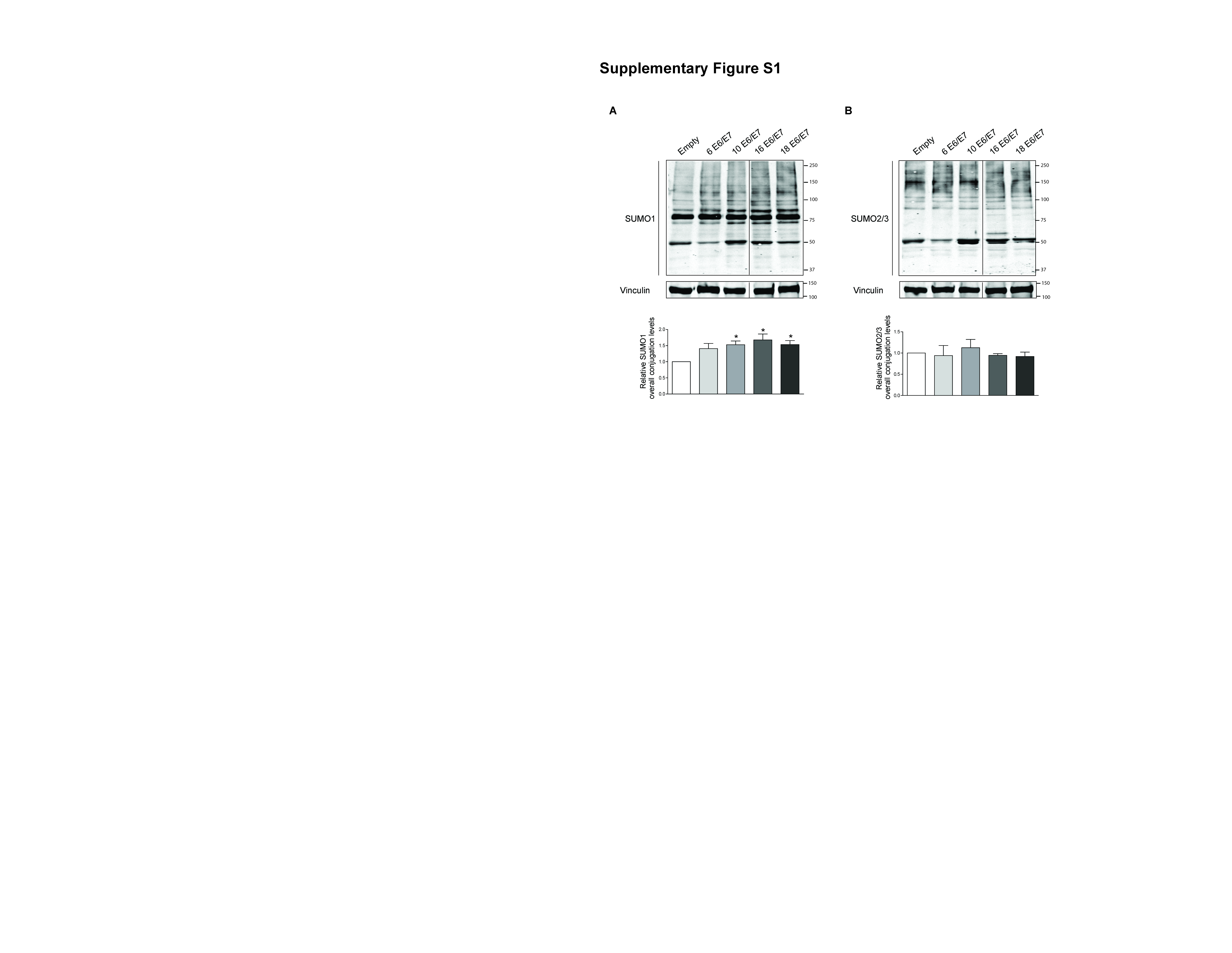


**Figure B. HPV E6/E7s from diverse HPV types preferentially promote SUMO1 conjugation *in vitro*.** Top: Representative WB of overall SUMO1 **(A)** and SUMO2/3 **(B)** conjugation levels in HKs transduced with the indicated recombinant retroviruses. Bottom: quantification of SUMO1 (left) and SUMO2/3 (right) overall conjugation as normalized to Vinculin levels. Data are expressed as fold over the empty-transduced HKs. Bars represent means ± SEM of n=3 different donors.*P<0.05 (Kruskal–Wallis one-way ANOVA with Dunn’s post hoc test) compared with empty control groups.


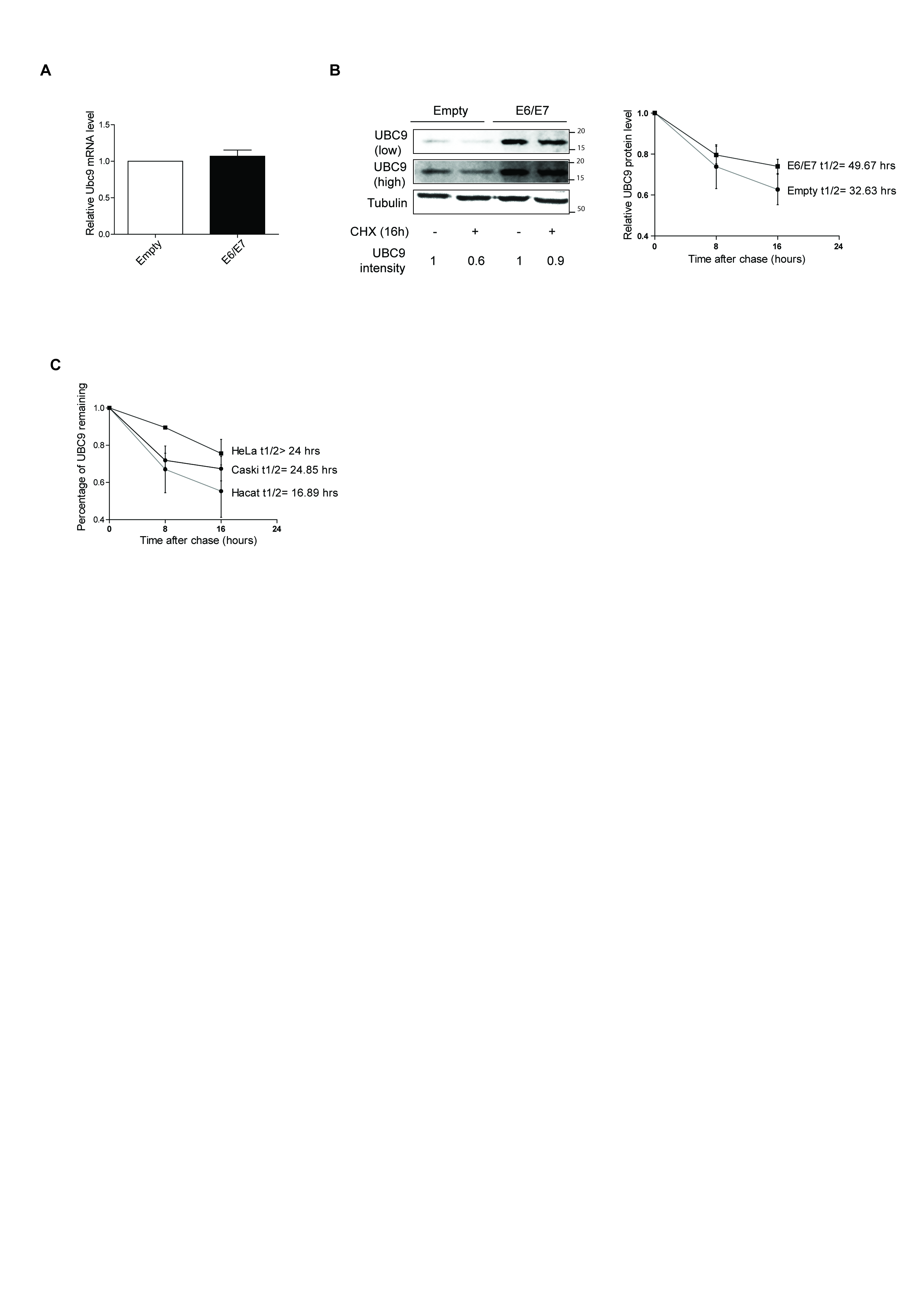


**Figure C. UBC9 overexpression by HPV16 E6/E7 occurs at post-translational level.** RT-qPCR analysis of UBC9 expression in empty or HPV16 E6/E7-expressing HKs using specific primers listed in Supplementary Table S1. Data are expressed as fold over the empty-transduced HKs. Bars represent means ± SEM of n=14 different biological replicates.


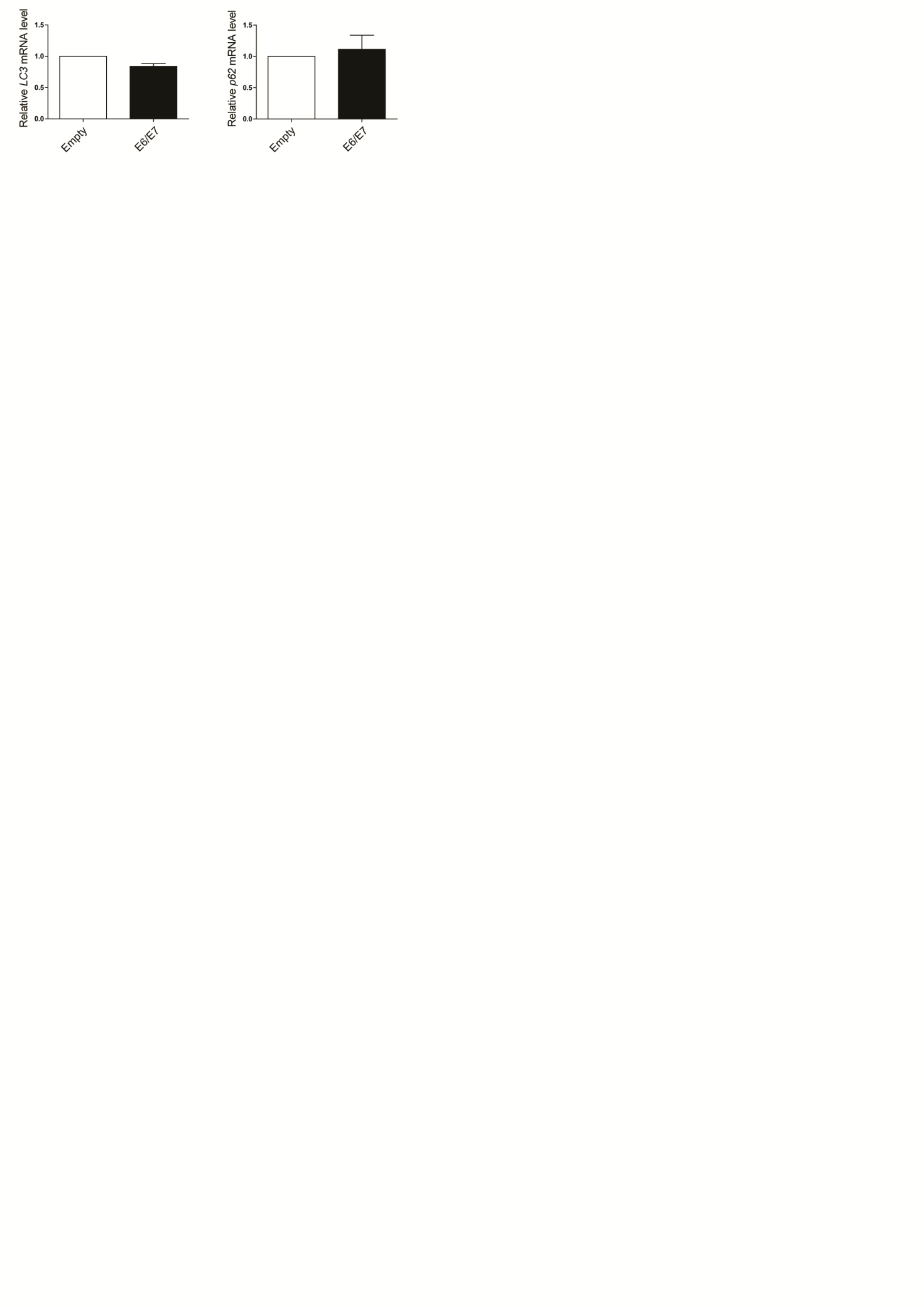


**Figure D. HPV16 E6/E7 do not affect LC3 and p62 transcription.** RT-qPCR analysis of *LC3* (left) and *p62* (right) expression in empty or HPV16 E6/E7-expressing HKs using specific primers listed in Supplementary Table 1. Data are expressed as fold over the empty-transduced HKs. Bars represent means ± SEM of n=5 different donors.


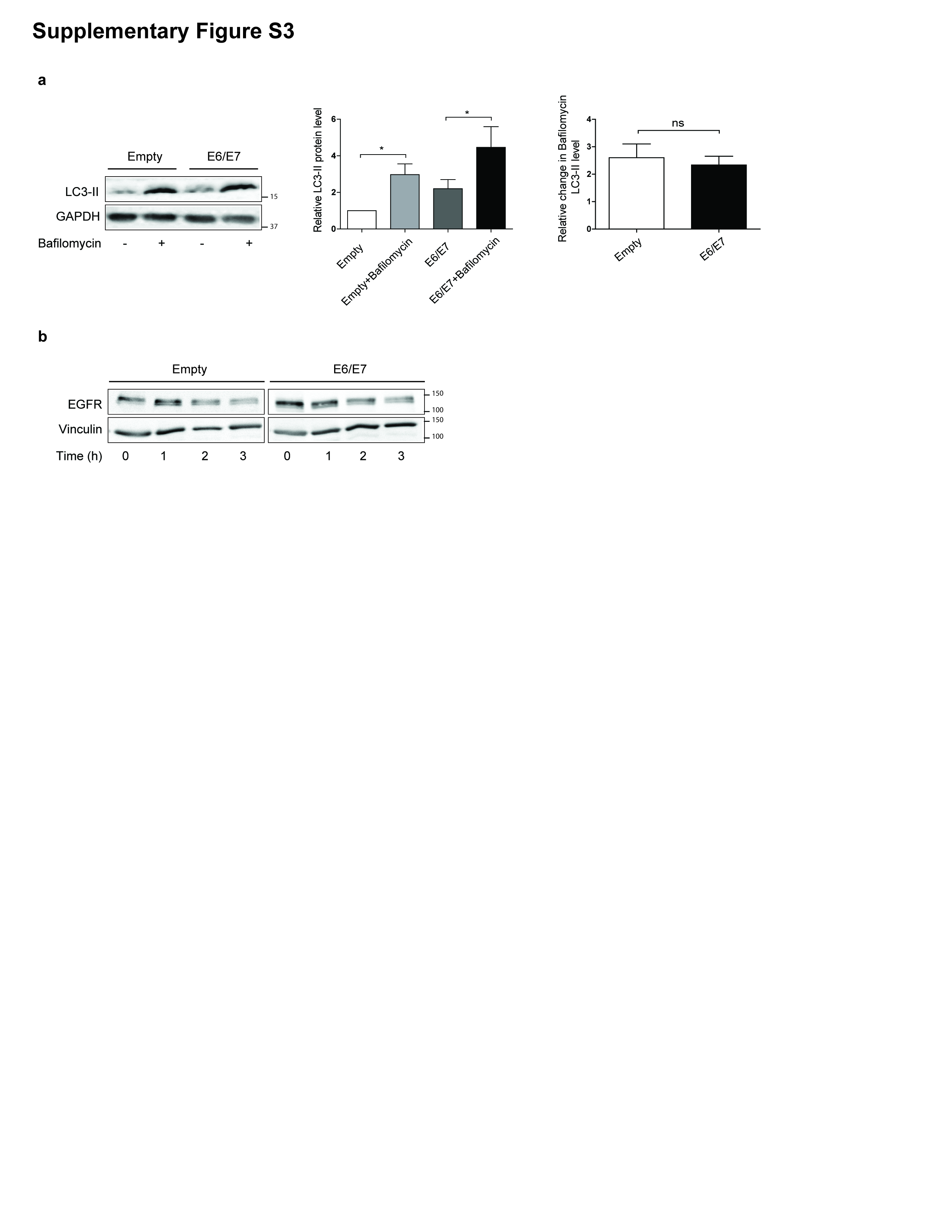


**Figure E. Lysosomal degradation is not affected by HPV16 E6/E7. (a)** Left: Representative WB analysis of bafilomycin-induced change in LC3-II levels. Cells were treated with bafilomycin for 8 hours. Middle: Densitometric quantification of LC3-II protein bands intensities normalized to GAPDH levels. Data are expressed as fold over empty cells. Bars represent means ± SEM in n=9 different biological replicates. *P < 0.01; ns: not significant (Kruskal–Wallis one-way ANOVA with Dunn’s post hoc test). Right: Autophagic flux was measured by dividing the LC3-II band intensities of bafilomycin-treated cells from the untreated cells. Data are expressed as densitometric arbitrary units normalized to GAPDH levels. Bars represents means ± SEM of n= 9 different biological replicates. ns: not significant (independent-sample t-test). **(b)** Representative WB analysis of EGFR degradation in empty and HPV16 E6/E7 HKs. EGFR is similarly degraded in both control and HPV16 E6/E7 transduced cells. n=3.


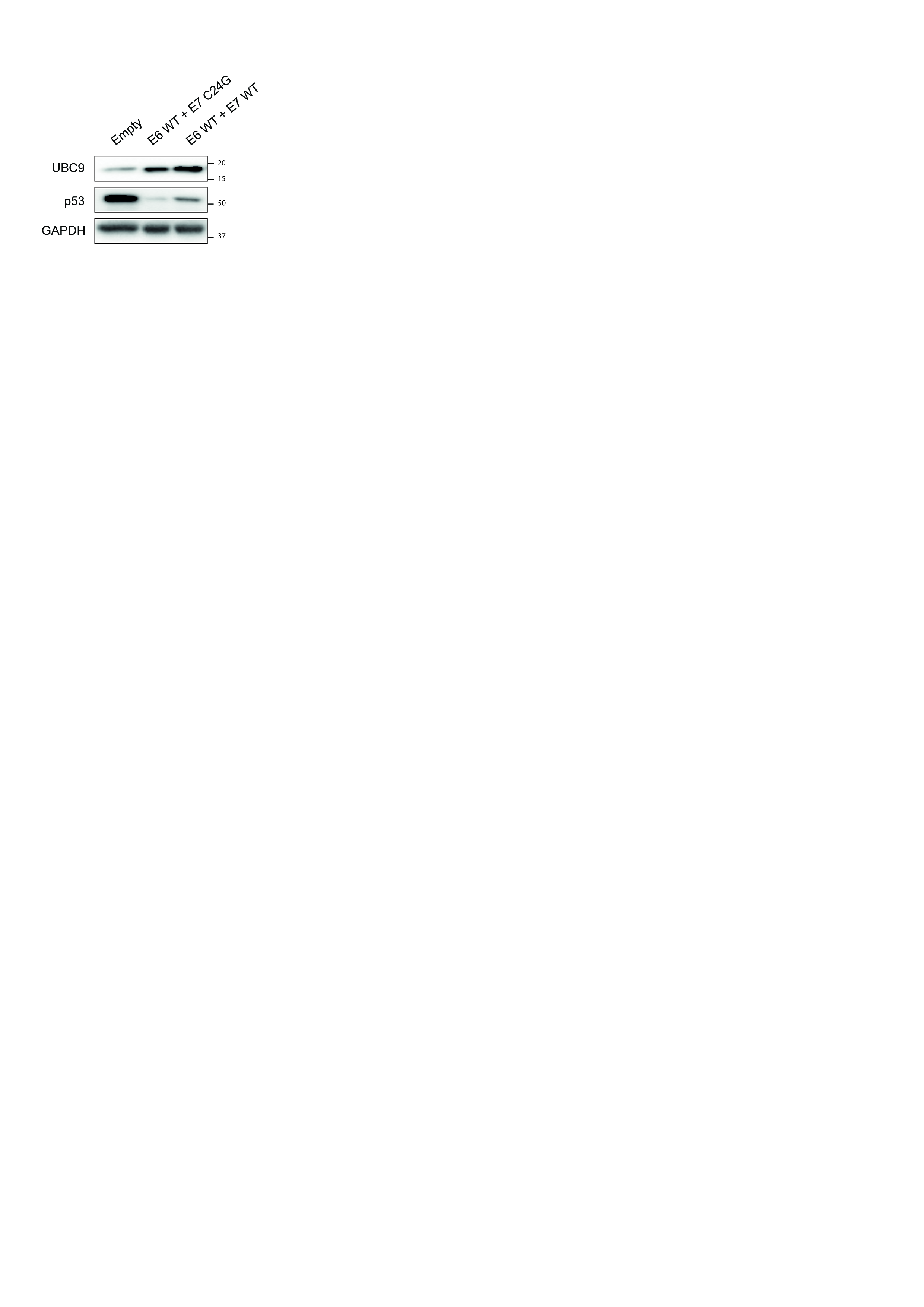


**Figure F. E7 pRb binding mutant partially reduced E6 WT ability to promote UBC9 up-regulation.** Representative WB analysis of HKs transduced with the indicated recombinant retroviruses and blotted with anti-UBC9 antibody. p53 is reported as control for E6 and E7 expression.


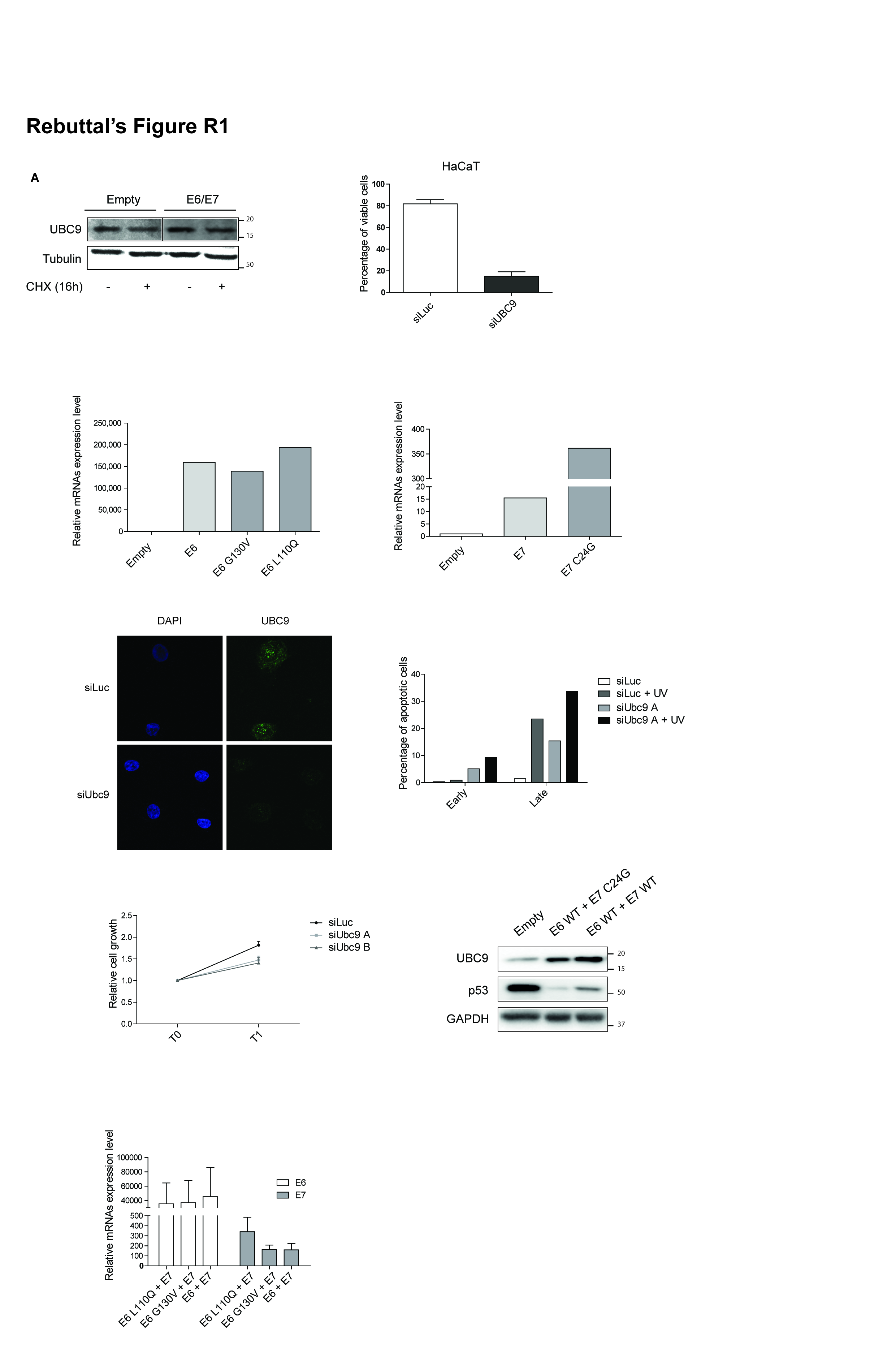


**Figure G. Relative mRNAs expression levels of E6/E7 mutants in HKs.** RT-qPCR analysis of viral proteins expression in HKs using specific primers listed in Supplementary Table 1. Data are expressed as fold over the empty-transduced HKs. Bars represent means ± SEM of at least n=3 different donors.

**Table A. Primers used in this study.**

| Primer | Sequence |
| --- | --- |
| 6E6 FOR  6E6 REV | 5’- atgcactgaccacagcagag -3’  5’- gcggtttgtgacacaggtag -3’ |
| 6E7 FOR  6E7 REV | 5’- atgaggtggacgaagtggac -3’  5’- cgcagatgggacacactatg -3’ |
| 10E6 FOR  10E6 REV | 5’- GGCATTGTACGACGCCTAA -3’  5’- GGTCTCCTCTTCCACACCTTC -3’ |
| 10E7 FOR  10E7 REV | 5’- GAACCAGCACAACAAGCGTA -3’  5’- TTATATCTGCGTGGCTGCAC -3’ |
| 11E6 FOR  11E6 REV | 5’- GAAAGGCACGCTTCATAAAACT -3’  5’- TTAGGGTAACAAGTCTTCCATGC -3’ |
| 11E7 FOR  11E7 REV | 5’- TGGAAGACTTGTTACCCTAAAGGA -3’  5’- GCTCATAGCAATGTAACCCTACAG -3’ |
| 16E6 FOR  16E6 REV | 5’- ATGTTTCAGGACCCACAGGA -3’  5’- CAGCTGGGTTTCTCTACGTGTT -3’ |
| 16E7 FOR  16E7 REV | 5’- CAGAGGAGGAGGATGAAATAGATGG -3’  5’- CACAACCGAAGCGTAGAGTCACAC -3’ |
| 18E6 FOR  18E6 REV | 5’- gaaaaacgacgatttcacaaca -3’  5’- ctcggttgcagcacgaat -3’ |
| 18E7 FOR  18E7 REV | 5’- gaaagctcagcagacgacct -3’  5’- cacaaaggacagggtgttca -3’ |
| SV40 LT FOR  SV40 LT REV | 5’- GACTCAGGGCATGAAACAGG-3’  5’- ACTGAGGGGCCTGAAATGA-3’ |
| UBC9 FOR  UBC9 REV | 5’- ACAGTGTGCCTGTCCATCTTAGAG -3’  5’- TGTTTTGGCAGTAAATCGTGTAGG-3’ |
| LC3 FOR  LC3 REV | 5’- CATGAGCGAGTTGGTCAAGA -3’  5’- CCATGCTGTGCTGGTTCA -3’ |
| p62 FOR  p62 REV | 5’- AGCTGCCTTGTACCCACATC -3’  5’- CAGAGAAGCCCATGGACAG -3’ |

**Table B. siRNAs sequences.**

| siRNA | dsRNA oligonucleotide sequence |
| --- | --- |
| siLuc | 5’- CGUACGGGGAAUACUUCGA -3’ sense  5’- UCGAAGUAUUCCCCGUACG -3’ antisense |
| siUbc9 A | 5’- UCGAACCACCAUUAUUUCACCCGAA -3’ sense  5’- UUCGGGUGAAAUAAUGGUGGUUCGA -3’ antisense |
| siUbc9 B | 5’- GCUCAAGCAGAGGCCUACACGAUUU -3’ sense  5’- AAAUCGUGUAGGCCUCUGCUUGAGC -3’ antisense |
| siE6/E7 A | 5’-CUUCGGUUGUGCGUACAAAGC -3’ sense  5’- GCUUUGUACGCACAACCGAAG-3’ antisense |
